# Advanced bioactive nanomaterials for biomedical applications

**DOI:** 10.1002/EXP.20210089

**Published:** 2021-12-28

**Authors:** Yu Zhao, Zhanzhan Zhang, Zheng Pan, Yang Liu

**Affiliations:** ^1^ Key Laboratory of Functional Polymer Materials of Ministry of Education State Key Laboratory of Medicinal Chemical Biology Frontiers Science Center for New Organic Matter College of Chemistry Nankai University Tianjin P. R. China

**Keywords:** bioactive nanomaterials, biomedical applications, nanotopography, physical structure, surface properties

## Abstract

Bioactive materials are a kind of materials with unique bioactivities, which can change the cellular behaviors and elicit biological responses from living tissues. Bioactive materials came into the spotlight in the late 1960s when the researchers found that the materials such as bioglass could react with surrounding bone tissue for bone regeneration. In the following decades, advances in nanotechnology brought the new development opportunities to bioactive nanomaterials. Bioactive nanomaterials are not a simple miniaturization of macroscopic materials. They exhibit unique bioactivities due to their nanoscale size effect, high specific surface area, and precise nanostructure, which can significantly influence the interactions with biological systems. Nowadays, bioactive nanomaterials have represented an important and exciting area of research. Current and future applications ensure that bioactive nanomaterials have a high academic and clinical importance. This review summaries the recent advances in the field of bioactive nanomaterials, and evaluate the influence factors of bioactivities. Then, a range of bioactive nanomaterials and their potential biomedical applications are discussed. Furthermore, the limitations, challenges, and future opportunities of bioactive nanomaterials are also discussed.

## Graphical abstract

In this review, we summary the recent advances of bioactive nanomaterials, and discuss the influence factors of bioactivities including the physical structure of bioactive nanomaterials, surface properties, and nanotopography. Then, a range of bioactive nanomaterials, including inorganic nanomaterials, carbon‐based nanomaterials, polymeric nanomaterials, and supramolecular‐based nanomaterials are discussed. In addition, we also introduce several typical applications of bioactive nanomaterials, including wound healing, cancer therapy, neurodegenerative disease therapy, and biocatalyst.

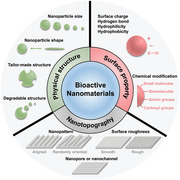

## 1 INTRODUCTION

Bioactive materials, which can induce biological responses upon interacting with proteins, cells, or tissues in vivo, have received considerable attentions in recent years.^[^ ^1^ ^]^ Compared with traditional biomaterials, a significant characteristic of bioactive materials is their bioactivities.^[^ ^2^, ^3^, ^4^, ^5^, ^6^ ^]^ In general, the bioactivities provided by bioactive materials include the capability of bonding hard or soft tissues,^[^ ^7^ ^]^ stimulating cell adhesion, differentiation, and proliferation,^[^ ^8^, ^9^, ^10^, ^11^ ^]^ mimicking the bio‐matrix for tissue regeneration,^[^ ^12^ ^]^ recognizing specific proteins and/or cells for biomimetics, releasing bioactive ions or molecules,^[^ ^13^, ^14^ ^]^ catalytic activities,^[^ ^15^, ^16^ ^]^ and targeted drug delivery.^[^ ^17^ ^]^ With these properties, bioactive materials show great potentials of changing cellular behaviors and functions and eliciting specific responses from living tissues for diagnostics, therapeutics, and regenerative medicine.^[^ ^18^ ^]^

In the late 1960s, the bioactive material was initially discovered by Larry Hench. Hench found that bioglass exhibited the capability to react with surrounding bone tissue to form a strong interfacial bond. Then, he defined the concept of the bioactive materials.^[^ ^19^ ^]^ By definition, bioactive materials are a class of biomaterials that can induce the biological response at the interface of materials via formation of a strong interaction or bond between materials and surrounding tissues.^[^ ^20^ ^]^ The discovery of bioactive materials results in an epochal shift in the perspectives regarding the reactivity at the material‐tissue interfaces. Thereafter, researchers began to realize that the materials did not necessarily involve a risk to human bodies if the products of these reactions were beneficial, and might hold great promise for biomedical applications.^[^ ^21^ ^]^ For example, the researchers from different groups have found that by changing the composition of bioactive glasses, or combining bioactive glasses with inorganic materials, metal or polymers, various bioactive materials can be constructed and exhibit excellent bioactivities for tissue repair and regeneration.^[^ ^22^, ^23^ ^]^ Before that, one requirement of biomaterials was that the material should not react with cells and/or tissues, because the only tissue responses we knew at that time were the uncontrollable inflammation and foreign body reactions.^[^ ^24^, ^25^ ^]^ By 1999, the biomaterial was redefined as “a material intended to interface with biological systems to evaluate, treat, augment, or replace any tissue, organ, or function of the body.” It means that there is an increasing trend for biomaterials to shift from traditional biomaterials to the bioactive materials.^[^ ^26^, ^27^ ^]^

With the development of material science and nanotechnology, the definition of bioactive material has been extended well beyond the scope proposed by Hench. Advances in nanotechnology have led to the development of advanced bioactive nanomaterials with customized properties.^[^ ^28^, ^29^, ^30^, ^31^ ^]^ As an important subclass of biomaterials, bioactive nanomaterials are not a simple miniaturization of the macroscopic materials. They exhibit unique bioactivities due to their nanoscale size, high specific surface area, and precise nanostructure, which significantly influence the interactions between materials and biological systems.^[^ ^32^ ^]^ For example, design of the architectures with nanoscale precision has demonstrated its tremendous potentials in regulating cellular behaviors, including cell adhesion, differentiation, proliferation, and recognition.^[^ ^33^ ^]^ In other studies, bioactive nanomaterials exhibit excellent capability to interact with proteins, nucleic acids, saccharides, signaling molecules, as well as the complex extracellular matrices (ECMs).^[^ ^34^, ^35^, ^36^ ^]^ In view of their physicochemical properties and bioactivities listed above, bioactive nanomaterials are expected to provide a novel platform for personalized medicine, which may change the future shape of the pharmaceutical industry. In addition, bioactive nanomaterials can also act as carriers to enhance the efficacy and precision by delivering therapeutic or diagnostic agents to cells and tissues.^[^ ^17^, ^37^, ^38^, ^39^ ^]^

It is worthy to note that the definition of bioactive nanomaterials and responsive nanomaterials are different. Bioactive nanomaterials are an important class of nanomaterials, which can induce biological response upon interacting with proteins, cells, or tissues. Most of the bioactive nanomaterials can regulate cellular behaviors and functions and elicit specific responses in living tissues. In contrast, responsive nanomaterials can response to various bio‐relevant stimuli (e.g., tissue‐specific pH, redox potentials, and enzyme types and concentrations), as well as the external stimuli (e.g., light exposure and heat). Upon the stimuli, responsive nanomaterials change their own structures in response to these stimuli, resulting in the change of the physicochemical properties of the materials (e.g., the surface charge, exposure of the cell‐penetrating peptide or cell‐targeting ligand, and control of drug release). Nowadays, responsive nanomaterials have been widely used to construct the smart drug delivery systems.

**SCHEME 1:**
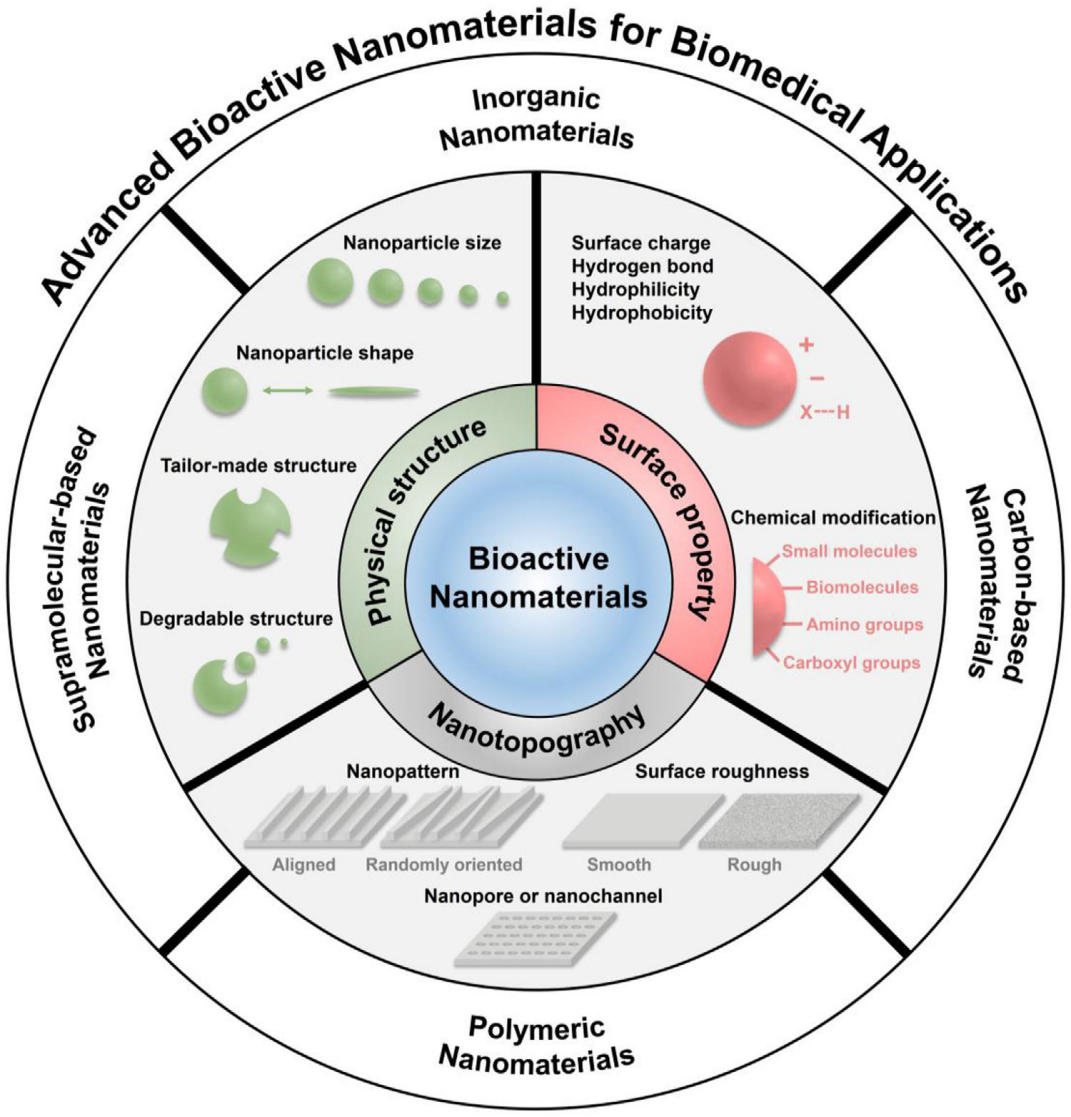
Advanced bioactive nanomaterials for biomedical applications

At present, the applications of bioactive nanomaterials have covered many important medical fields. However, the bioactive nanomaterials and the influence factors of bioactivities have not yet been systematically summarized. In this review, we summarize the recent advances of bioactive nanomaterials, and discuss the influence factors of bioactivities including the physical structure of bioactive nanomaterials, surface properties, and nanotopography. Then, a range of bioactive nanomaterials, including inorganic nanomaterials, carbon‐based nanomaterials, polymeric nanomaterials, and supramolecular‐based nanomaterials are discussed (Scheme 1). In addition, we also introduce several typical applications of bioactive nanomaterials, including wound healing, cancer therapy, neurodegenerative disease therapy, and biocatalyst. Finally, the future directions and challenges of bioactive nanomaterials are discussed.

## 2 BIOACTIVE NANOMATERIALS

Nanomaterials are the materials that their dimensions are reduced to the nanoscale. Compared with traditional bioactive materials, the bioactive nanomaterials typically have well‐defined architecture and surface property.^[^ ^32^ ^]^ The architectures and surface properties have a significant influence on the interactions between the nanomaterials and biological systems.^[^ ^1^ ^]^ Moreover, the presence of the nanoscale precision on bioactive nanomaterials (e.g., nanopattern, nanopore, nanochannel) can create a biomimetic feature towards proteins, resulting in regulation of cellular behaviors.^[^ ^40^ ^]^ In this section, the factors influencing the bioactivity of nanomaterials are discussed. The bioactive nanomaterials with controllable bioactivity, which are based on biological recognition, are also discussed. Finally, different types of bioactive nanomaterials for biomedical applications are introduced.

### 2.1 Influencing factors on bioactivity

**FIGURE 1:**
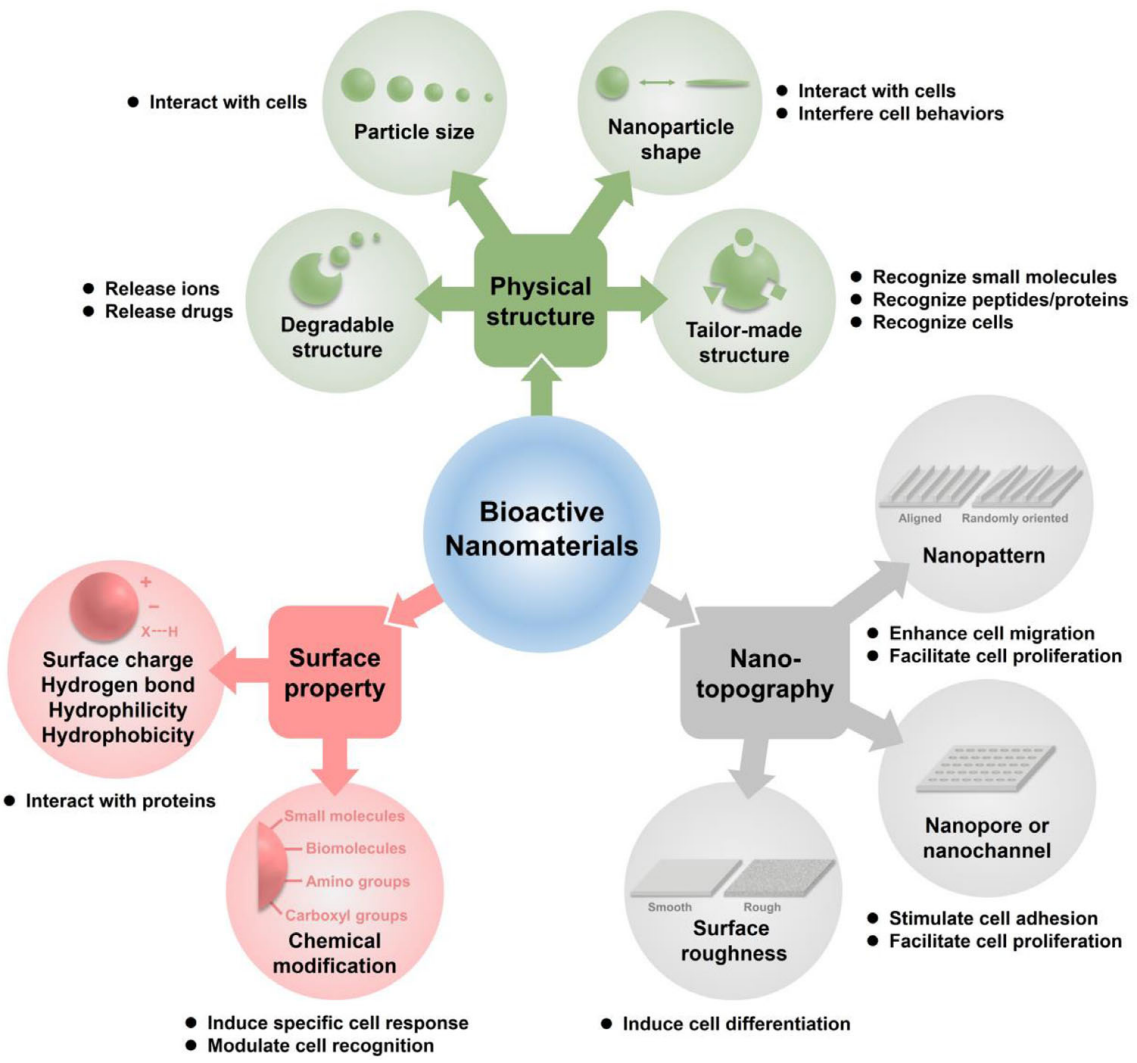
The influence factors of bioactivities

The bioactivities of bioactive nanomaterials are influenced by numerous factors. The physical structure of materials, surface property, and nanotopography, which have effects on the interactions between nanomaterials and biological systems are the key factors requiring consideration (Figure 1). In addition, the factors which can control the release of bioactive ions or molecules from bioactive nanomaterials should also be considered.

#### 2.1.1 Physical structure

The particle size and the structure of nanomaterials significantly affect their bioactivities.^[^ ^41^, ^42^ ^]^ When particle size decreases, a greater proportion of atoms or molecules can be found on the surface of nanoparticle. Research shows that a 30 nm sized particle has 5% of its atoms or molecules on its surface, and a 3 nm sized particle has 50% of its atoms or molecules on the surface. Therefore, nanoparticles have much greater specific surface areas than the particles with larger size.^[^ ^43^ ^]^ Considering that numerous biological reactions occur at surfaces and interfaces, it means that the materials in the form of nanoparticle have much higher activity than the same mass of materials with larger particle size. Yang et al. prepared a series of size‐controlled hydroxyapatite nanoparticles (denoted as Nano‐Haps), and evaluated their size effects on human osteoblast‐like MG‐63 cells. The results indicated that the proliferation of MG‐63 cells was closely related to the particle size of Nano‐Haps. Nano‐Haps with the size of 20 nm had the prominent effects on promoting cell growth and inhibiting cell apoptosis.^[^ ^44^ ^]^ Silver nanoparticles (Ag NPs) are well‐known antimicrobial agents, and have been widely used in wound dressings and coatings and in medical devices. Jong et al. found that particle size played a crucial role in determining these effects. In this study, Ag NPs with different particle sizes including 20, 80, and 113 nm were employed, and their cytotoxicity was evaluated, respectively. They found that 20 nm sized Ag NPs with higher specific surface area were more toxic than the larger nanoparticles and silver ions.^[^ ^45^ ^]^ These results suggested the key role of particle size in affecting the bioactivity of nanomaterials.

Besides the particle size, the structure of nanomaterials has a great influence on their bioactivities. For example, molecularly imprinted nanoparticles (MINPs) can recognize and bind the targeted biomolecules (e.g., peptides, proteins) with a high affinity and selectivity, which show great potentials of acting as synthetic chemical receptors.^[^ ^46^, ^47^ ^]^ The bioactivity of MINPs is highly dependent on their tailor‐made nanostructures.^[^ ^48^ ^]^ To obtain a MINP, various types of monomers including hydrogen‐bonding, positively charged, negatively charged and hydrophobic monomers are polymerized in the presence of the targeted molecules.^[^ ^49^, ^50^ ^]^ The collective interactions between monomers and targeted molecule during polymerization lead to the formation of complementary binding sites in the obtained MINPs, thereby achieving a tailor‐made structure. Thus, the obtained MINPs can recognize the targeted molecule via a combination of multiple hydrogen‐bonding, electrostatic and hydrophobic interactions at the complementary three‐dimensional interface. Nowadays, MINPs have been developed to target and visualize protein/glycoprotein‐based cell receptors overexpressed in certain diseases, such as tumors.^[^ ^51^ ^]^ Guo et al. demonstrated an MINP that could recognize and bind testosterone, thereby blocking the testosterone‐androgen receptor pathway for prostate cancer treatment.^[^ ^52^ ^]^ Shea et al. reported an MINP capturing vascular endothelial growth factor (VEGF), thereby suppressing the growth of tumors via reducing angiogenesis.^[^ ^53^ ^]^ Liu et al. developed a boronate‐based MINP that could target human epidermal growth factor receptor‐2 (HER2)^+^ breast tumor cells through binding the glycans on HER2, thereby inhibiting tumor growth via blocking HER2‐dependent signaling pathway.^[^ ^54^ ^]^ These results indicated that the tailor‐made structure played a key role in affecting the bioactivity of MINPs.

The morphological structure of nanomaterials is also closely correlated with their bioactivities, which shows great potentials to interfere the cellular behaviors. For example, Lam et al. developed a transformable peptide that could self‐assemble into nanoparticles, however, these particles transformed into nanofibers when binding to the HER2 on the surface of tumor cells. As a result, the nanofibers disrupted dimerization of HER2 and subsequent downstream signal, thereby inhibiting growth of tumor cells.^[^ ^55^ ^]^ In addition, researchers also found that the carbon nanofibers of 60 nm in diameter could effectively increase osteoblast adhesion and simultaneously decrease competitive cell adhesion (e.g., fibroblast cell and smooth muscle cell), thereby stimulating sufficient osseointegration. Similar results were also observed from carbon nanotubes.^[^ ^56^, ^57^, ^58^ ^]^ All of these results indicated that nanomaterials with different structure are capable of exhibiting different bioactivities.

Nanomaterials with degradable structure also show the bioactivities by releasing bioactive ions or molecules. Metal‐organic frameworks (MOFs) are a class of inorganic‐organic hybrid nanomaterials built from metal ions bridged by organic linkers.^[^ ^59^, ^60^ ^]^ MOFs can degrade rapidly and release their metal ions or organic linkers in acid conditions. Zhang et al. developed a bioactive MOFs (denoted as Zn_2_(ppa)_2_(1,3‐bdc)(H_2_O)), which was composed of Zn(II), dicarboxylate ligand, and pipemidic acid (Hppa). Zinc is an essential micronutrient for the life, which has been widely used as an antimicrobial agent. Hppa is a gyrase inhibitor, which has a broad clinical application against enteric and urinary tract infections.^[^ ^61^ ^]^ As a result, Zn_2_(ppa)_2_(1,3‐bdc)(H_2_O) released the Hppa, as well as Zn(II) under the acidic condition, which showed great potentials of combating various pathogenic bacterial species.^[^ ^62^ ^]^ Recently, Shi et al. reported a voriconazole‐inbuilt zinc 2‐methylimidazolates framework (denoted as V‐ZIF), in which the voriconazole was employed as a building block to construct the bioactive MOF. V‐ZIF significantly reduces the leakage of voriconazole. Effective release of voriconazole was achieved by dissociating voriconazole from the MOF in the acidic condition, such as in biofilms.^[^ ^13^ ^]^ In open wounds infected by *C. albicans*, V‐ZIF exhibited effective antifungal performance, thereby accelerating wound closure. In addition, metal‐containing nanomaterials such as metal oxide nanoparticles and metal ions doped inorganic nanoparticles also have attracted enormous interest in recent years. They exhibit their bioactivities by releasing metal ions. For example, Zheng et al. demonstrated an iron‐based nanomaterial that can release bioactive Fe^2+/3+^ and trigger Fenton reaction to interfere with the biological processes associated with cell death.^[^ ^63^, ^64^ ^]^ Waldman et al. found that Ag NPs could release Ag^+^ persistently in the bacteria, thereby making their bactericidal activity more durable and effective.^[^ ^65^, ^66^ ^]^

#### 2.1.2 Surface property

The surface properties of nanomaterials have critical influence on their bioactivities.^[^ ^67^ ^]^ The responses of biological systems to nanomaterials are closely associated with the surface properties of nanomaterials.^[^ ^68^, ^69^, ^70^ ^]^ Most bioactive nanomaterials interact with biological systems via ligand‐receptor binding pathways, as well as non‐specific adhesions. In general, there are two main design approaches in controlling the surface properties of bioactive materials. First, the surface property of nanomaterials (e.g., the surface charge and the hydrophilicity/hydrophobicity) is adjusted to a specific state, thereby achieving an ideal surface bioactivity. For example, Chen et al. found that the gold nanoparticles (Au NPs) with negatively charged surface (zeta potential, −38 mV) could effectively inhibit amyloid‐β (Aβ) fibrillization and induce Aβ to form the less toxic species. In contrast, the positively charged Au NPs (+7 mV) had no effect on interfering the aggregation process of Aβ proteins.^[^ ^71^ ^]^ Wang et al. reported a biocompatible and biodegradable polymer nanoparticle, which was constructed by poly(lactic‐co‐glycolic acid) (PLGA). The PLGA nanoparticle could bind to tumor antigens (a class of tumor‐specific proteins) via non‐covalent hydrophobic‐hydrophobic interactions. As a result, PLGA nanoparticle captured and delivered tumor antigens into antigen‐presenting cells (APCs) after irradiation treatment, resulting in the enhanced efficacy of immune checkpoint blockade (ICB) immunotherapy in B16F10 melanoma‐bearing mice. In this sense, tumor cells or tissue are converted into in situ vaccines by the PLGA nanoparticles.^[^ ^72^ ^]^ Heat shock proteins (HSPs) have proved to be an efficient immune stimulator to combat various types of tumors, including melanoma, glioblastoma, and pancreatic cancer.^[^ ^73^ ^]^ HSPs capture the tumor‐associated antigens via their microdomains and deliver the antigens into the antigen presenting cells (APCs), thereby triggering robust antitumor responses. Inspired by HSPs, Shi et al. recently demonstrated a mixed‐shell micelle (denoted as nChap) with surface hydrophobic microdomains to mimic HSPs for cancer immunotherapy. In this design, nChap captured tumor antigens via hydrophobic‐hydrophobic interactions and delivered antigens into APCs. More importantly, nChap could escape from the lysosomes through transforming the surface hydrophobic microdomains into the positively charged ones, thereby enhancing the cross‐presentation of tumor antigens in cytoplasm. As a result, nChap triggered robust T cell‐dependent antitumor responses in melanoma bearing mice.^[^ ^74^ ^]^

Another commonly used approach for achieving an ideal surface property is chemical modification. By directly immobilizing bioactive ligands including small molecules, peptides, and antibodies on the surface of materials, the obtained bioactive nanomaterials show the potential to induce a specific cellular response.^[^ ^67^ ^]^ The reactive amino (–NH_2_) and carboxyl (–COOH) groups are usually employed as the coupling sites for covalent attachment of these ligands onto the material surface, thereby achieving a bioactive nanosurface. For example, Tao et al. reported a bioactive nanoinhibitor by conjugating mesenchymal‐epithelial transition (MET)‐targeting peptides on a polymeric nanoparticle. The binding affinity of nanoinhibitor to MET factor increased 3 orders of magnitude to 1.32 × 10^−10^ M, compared with those of free peptides (*K*_D_ = 3.96 × 10^−7^ M). As a result, this nanoinhibitor efficiently attenuated the proliferation and invasion of glioblastoma U87MG cells through blocking MET signaling.^[^ ^75^ ^]^ Wang et al. demonstrated an anti‐IgG (Fc specific) antibody modified nanoparticle, which was prepared by conjugating multiple anti‐IgG antibodies onto a nanoparticle surface (denoted as αFc‐NP). The αFc‐NP, as a versatile antibody immobilization platform, could efficiently immobilize two types of monoclonal antibodies via Fc‐specific noncovalent binding for cancer immunotherapy. Compared with the covalent conjugation, noncovalent immobilization of these monoclonal antibodies did not damage their antigen‐binding activities. They chose two types of immune checkpoint inhibitors including anti‐PD1 antibody and anti‐PDL1 antibody as model monoclonal antibodies, and found that this formulation could effectively promote T cell‐tumor cell interaction and trigger robust T cell‐dependent antitumor responses.^[^ ^76^ ^]^ Moreover, they also validated the potential of this approach in macrophage‐ and natural killer cell‐mediated antitumor immune responses in vivo.

#### 2.1.3 Nanotopography

Many experimental observations have shown that the cells exhibited various behaviors on the substrates with different nanotopographies, indicating that cells could distinguish the geometry of the substrates (e.g., nanopattern and surface roughness).^[^ ^77^, ^78^, ^79^ ^]^ For example, Li et al. prepared an aligned nanofibrous scaffold through immobilizing extracellular matrix proteins (ECM) and growth factors onto the surface of nanofibers. In this study, the nanofibrous scaffolds were constructed and employed to simulate the physical and biochemical properties of native ECM. The results indicated that aligned nanofibrous scaffolds could effectively enhance skin cell migration and induce neurite outgrowth during wound healing compared to the randomly oriented nanofibrous scaffolds.^[^ ^80^ ^]^ Fu et al. demonstrated the influence of surface roughness on embryonic stem cell adhesion. They found that the undifferentiated cells preferentially adhered to the smooth surfaces, rather than the rough surfaces. Moreover, smooth surface could maintain the self‐renewal capacity of ESCs, while rough surface induced the differentiation of ESCs.^[^ ^40^ ^]^ These studies revealed the importance of nanotopology in guidance of cell behaviors. In addition, the substrates with nanopore or nanochannel structure also exhibit unique bioactivities. The high porosity and pore interconnectivity of substrates have an important effect on cell proliferation and adhesion. For example, the porous nanostructure can facilitate the adequate transport of nutrients and cellular waste products, thereby providing a better environment for cell growth.^[^ ^81^, ^82^ ^]^

#### 2.1.4 Others

The chemical composition and inherent structure of nanomaterials also have influences on their bioactivities, especially the catalytic activities.^[^ ^83^, ^84^, ^85^ ^]^ The catalytic sites of natural enzymes normally involve a multivalent metal ion such as Fe^2+^/Fe^3+^ and Cu^+^/Cu^2+^. By mimicking the catalytic sites of natural enzymes, various metal or metal oxide‐based nanomaterials have been served as promising substitutes of traditional enzymes (denoted as nanozymes).^[^ ^86^ ^]^ For example, ferromagnetic (Fe_3_O_4_) nanoparticles and some noble metal‐based nanoparticles (e.g., gold, silver, palladium, platinum, and their hybrids) were found to mimic the enzyme behaviors of peroxidase and catalase (CAT).^[^ ^87^, ^88^ ^]^ Cuprous oxide (Cu_2_O) nanoparticles presented glucose oxidase, lactase, and cytochrome C oxidase‐mimicking properties.^[^ ^89^ ^]^ Manganomanganic oxide (Mn_3_O_4_) nanoparticles could mimic three cellular antioxidant enzymes including glutathione peroxidase, superoxide dismutase (SOD), and CAT.^[^ ^90^ ^]^ Additionally, MOFs also draw great attention as a kind of peroxidase‐mimicking nanomaterials, resulting from their framework flexibility and large surface areas.^[^ ^91^ ^]^ For example, by chelating Fe or Cu ions with organic linkers, catalytic sites are created to achieve optimal cooperativity for peroxidase reactions. More importantly, MOFs with a precise nanostructure can serve as an excellent model to explore and validate the catalytic activities of nanomaterials.

### 2.2 Types of bioactive nanomaterials

With the rapid development of material science, various types of bioactive nanomaterials for biomedical applications have been developed, including inorganic nanomaterials, polymeric nanomaterials, carbon‐based nanomaterials, and supramolecular nanomaterials (Table 1).

**TABLE 1:** Advanced bioactive nanomaterials and their biomedical applications

**Materials**	**Types**	**Bioactivities**	**Applications**	**Ref**.
Hydroxyapatite NP	Inorganic nanomaterials	Facilitate cell proliferation	Promotion of osteoblast‐like cell proliferation	^[^ ^44^ ^]^
CS/nHAp/nCu‐Zn scaffold	Inorganic nanomaterials	Facilitate cell proliferation, the capability to bind hard or soft tissues	Cell penetration and bone tissue formation	^[^ ^66^, ^99^ ^]^
Ag NP	Inorganic nanomaterials	Release ions	Antimicrobial agents	^[^ ^45^ ^]^
MSN‐GACs	Inorganic nanomaterials	Enhance cell adhesion	Hemostasis	^[^ ^136^ ^]^
Dopamine‐modified MSN	Inorganic nanomaterials	Promote cell migration	Wound healing	^[^ ^137^ ^]^
Iron‐based NP	Inorganic nanomaterials	Release ions	Ferroptotic cancer therapy	^[^ ^14^ ^]^
Bioglass NP	Inorganic nanomaterials	Release ions	Cancer immunotherapy	^[^ ^138^ ^]^
UCNP/ICG/RB‐mal	Inorganic nanomaterials	Controllable surface properties for antigen capture	Cancer immunotherapy	^[^ ^139^ ^]^
aCD47@CaCO_3_	Inorganic nanomaterials	Release ions	Cancer immunotherapy	^[^ ^140^ ^]^
Peptide conjugated Au NP	Inorganic nanomaterials	Catalytic activities	Cancer cell immunoassay	^[^ ^141^ ^]^
Fe_3_O_4_ MNP	Inorganic nanomaterials	Catalytic activities	Peroxidase‐like activity/Immunoassays	^[^ ^142^, ^143^ ^]^
BSA‐IrO_2_ NP	Inorganic nanomaterials	CT imaging capability	Cancer theranostics	^[^ ^144^ ^]^
Au NP	Inorganic nanomaterials	Controllable surface properties for biomimetics	Inhibition of Aβ fibrillization	^[^ ^71^ ^]^
BNNS@CuS	Inorganic nanomaterials		Detection of the total cholesterol in human serum	^[^ ^145^ ^]^
Zn_2_(ppa)_2_(1,3‐bdc)(H_2_O)	Inorganic‐organic hybrid nanomaterials	Release ions or molecules	Antimicrobial agents	^[^ ^62^ ^]^
V‐ZIF	Inorganic‐organic hybrid nanomaterials	Release ions or molecules	Biofilm‐associated infection treatment	^[^ ^13^ ^]^
MOF	Inorganic‐organic hybrid nanomaterials	Catalytic activities	Peroxidase‐mimicking nanomaterials	^[^ ^91^ ^]^
GOx/hemin@ZIF‐8	Inorganic‐organic hybrid nanomaterials	Catalytic activities	Detection of the glucose in drinks, blood, and urine	^[^ ^146^ ^]^
Az@MOF	Inorganic‐organic hybrid nanomaterials	Targeted delivery	Facilitating microglia‐mediated Aβ clearance	^[^ ^147^ ^]^
Magnetic MINP	Polymeric nanomaterials	Controllable surface properties for biomimetics	Prostate cancer treatment	^[^ ^52^ ^]^
MINP	Polymeric nanomaterials	Controllable surface properties for biomimetics	Inhibition of tumor growth via reducing angiogenesis	^[^ ^53^ ^]^
Boronate‐based MINP	Polymeric nanomaterials	Controllable surface properties for biomimetics	Inhibition of tumor growth via blocking HER2‐dependent signaling pathway	^[^ ^54^ ^]^
Nanoinhibitor	Polymeric nanomaterials	Controllable surface properties for changing cell behavior	Glioblastoma treatment	^[^ ^75^ ^]^
PLGA NP	Polymeric nanomaterials	Controllable surface properties for antigen capture	Cancer immunotherapy	^[^ ^72^ ^]^
nChap	Polymeric nanomaterials	Controllable surface properties for antigen capture	Cancer immunotherapy	^[^ ^74^ ^]^
αFc‐NP	Polymeric nanomaterials	Controllable surface properties for biomimetics	Cancer immunotherapy	^[^ ^76^ ^]^
Antibody‐like polymeric NP	Polymeric nanomaterials	Controllable surface properties	Cancer immunotherapy	^[^ ^111^ ^]^
mBiNE	Polymeric nanomaterials	Controllable surface properties	Cancer immunotherapy	^[^ ^109^, ^112^ ^]^
PC7A‐NP‐based nanovaccine	Polymeric nanomaterials	Controllable surface properties	Cancer immunotherapy	^[^ ^148^ ^]^
MINP	Polymeric nanomaterials	Controllable surface properties for biomimetics	Antidote	^[^ ^107^ ^]^
Aβ recognition element‐modified NP	Polymeric nanomaterials	Controllable surface properties	Attenuating Aβ‐induced neuron apoptosis	^[^ ^110^, ^149^ ^]^
MSPMs	Polymeric nanomaterials	Controllable surface properties	Inhibiting amyloid protein aggregation	^[^ ^150^, ^151^ ^]^
SWCNTs	Carbon‐based nanomaterials	Stimulate cell adhesion, induce cell differentiation	Cell adhesion and neurogenic differentiation of mesenchymal stem cells	^[^ ^115^ ^]^
MWCNTs	Carbon‐based nanomaterials	Induce cell differentiation	Osteogenic differentiation of mesenchymal stem cells	^[^ ^116^ ^]^
RGO‐PDA	Carbon‐based nanomaterials	Induce cell differentiation	Cell adhesion, proliferation, and osteogenic differentiation	^[^ ^119^ ^]^
Graphene oxide	Carbon‐based nanomaterials	Unclear	Activation of the autophagy of neurons and microglial cells	^[^ ^120^ ^]^
Platinum NPs/graphene oxide	Carbon‐based nanomaterials	Catalytic activities	Cancer cell detection	^[^ ^152^ ^]^
SWNTs‐based nanozyme	Carbon‐based nanomaterials	Catalytic activities	Treatment of methamphetamine (METH) addiction	^[^ ^153^ ^]^
Transformable peptide	Supramolecular‐based nanomaterials	Recognize specific proteins and/or cells, change cell behavior	Disrupting dimerization of HER2 for cancer treatment	^[^ ^55^ ^]^
Transformable peptide	Supramolecular‐based nanomaterials	Recognize specific proteins and/or cells for biomimetics	Trapping the bacteria by mimicking antimicrobial peptide human defensin‐6	^[^ ^130^ ^]^
AmpF peptide	Supramolecular‐based nanomaterials	Targeted drug delivery	Combinatorial chemo‐photodynamic therapy	^[^ ^17^, ^39^ ^]^
RADA16 peptide	Supramolecular‐based nanomaterials	Recognize specific proteins and/or cells for biomimetics	Hemostasis	^[^ ^154^ ^]^
Guanidinium‐modified pillar[5]arene	Supramolecular‐based nanomaterials	Biorecognition	Anti‐wound infections	^[^ ^155^ ^]^
Cyclodextrins and calixarenes	Supramolecular‐based nanomaterials	Biorecognition	Attenuating Aβ‐induced neuron damages	^[^ ^132^, ^156^ ^]^
Cyclodextrins and calixarenes	Supramolecular‐based nanomaterials	Biorecognition	Antidote	^[^ ^135^ ^]^

#### 2.2.1 Inorganic nanomaterials

Inorganic nanomaterials are a kind of nanomaterials with inorganic substances as the main body.^[^ ^92^ ^]^ The inorganic nanomaterials typically have better mechanical stability compared to organic or polymeric nanomaterials. During the last decade, various types of bioactive inorganic nanomaterials (e.g., silver, gold, platinum, iron, cobalt, titanium, silica, and ceramic particles) have been developed for regenerative medicine.^[^ ^93^, ^94^ ^]^ For example, an ideal bone graft substitute should be able to imitate the ECMs of natural bone for achieving good biocompatibility, and provide strong mechanical support for bone tissue regeneration. Inorganic nanomaterials are the most promising candidate as the bone graft substitutes due to their excellent mechanical strength.^[^ ^95^ ^]^ More importantly, the inorganic nanomaterials can maintain stability for several weeks in the body, thereby supporting the bone healing in the early stage of regeneration. For example, bioactive glasses, hydroxyapatite, nanosilicates, and silica nanoparticles have been widely used in bone tissue engineering. Among them, nanohydroxyapatite has been demonstrated to be of similar chemical composition and structure to the bone tissues. Thus, mesenchymal stem cells can effectively recognize and bind to nanohydroxyapatite, thereby facilitating the osteogenic differentiation.^[^ ^96^ ^]^ Metal‐based nanomaterials also show great potentials in bone tissue regeneration due to their antibacterial activity.^[^ ^97^, ^98^ ^]^ For example, Selvamurugan et al. reported a bio‐composite scaffold composed of chitosan (CS), nano‐hydroxyapatite (nHAp), and Cu‐Zn alloy nanoparticles (nCu‐Zn) for bone tissue engineering (denoted as CS/nHAp/nCu‐Zn scaffold). They found that combination of nano and micro arrangements provided an ideal interface for cell penetration and bone tissue formation. Compared with CS/nHAp scaffold, the addition of nCu‐Zn was accompanied by increased swelling behavior, decreased degradation, and increased antibacterial activity.^[^ ^66^, ^99^ ^]^ In addition, metal‐based materials also exhibited enzymatic activity to mimic the natural enzymes, which has been discussed in Section 2.1.4. Nowadays, the use of inorganic nanomaterials for applications in regenerative medicine has received much attentions. However, the toxicity, which is usually caused by the non‐specific and long‐term accumulation of inorganic nanomaterials in organs and normal tissues, is still a challenge and prevents them from clinical utilization.

#### 2.2.2 Polymeric nanomaterials

Polymeric nanomaterials have attracted numerous attentions in the biomedicine area due to their inherent biocompatibility and biodegradability.^[^ ^100^ ^]^ More importantly, they can be easily modified with active ligands for targeting the cells or tissues.^[^ ^101^, ^102^ ^]^ In recent years, various types of polymers have been used for the fabrication of polymeric nanomaterials for biomedical applications.^[^ ^103^, ^104^, ^105^ ^]^ For example, synthetic polymers are ideal candidates to design the MINPs with a tailor‐made structure or a customized surface for molecular recognition.^[^ ^46^ ^]^ As an important bioactive nanomaterial, polymeric MINPs can recognize and bind targeted biomolecules such as peptides and proteins with a high affinity and selectivity both in vitro and in vivo. The design and preparation of polymeric MINPs have been discussed in Section 2.1.1. At present, numerous MINPs have shown the great potentials as diagnostic and therapeutic agents. Sellergren et al. reported a sialic acid‐imprinted nanoparticle equipped with nitrobenzoxadiazole (NBD) fluorescent groups (denoted as AINP‐NBD) for bioimaging. AINP‐NBD displayed strong affinity for sialic acid, whereas binding of the competitor glucuronic acid and other monosaccharides were considerably weaker. As a result, AINP‐NBD selectively stained different tumor cell lines in correlation with the expression level of sialic acid.^[^ ^106^ ^]^ Shea et al. developed a polymer particle by optimizing the functional monomers used in MINP synthesis. The obtained polymer particle had a comparable binding affinity and selectivity for targeted molecule to those of natural antibodies. In this study, they prepared a MINP with strong affinity for melittin (*K*_dapp_ < 1 nM), a cytolytic peptide that was the basic component of bee venom. As a result, MINP captured melittin effectively in the bloodstream.^[^ ^107^ ^]^ The obtained melittin‐MINP complexes were then removed by the mononuclear phagocytic system, thereby diminishing the peripheral toxic symptoms and decreasing the mortality.

Polymeric nanoparticles with the capability of specifically recognizing targeted proteins or cells can also be synthetized by immobilizing bioactive ligands (e.g., small molecules, peptides, antibodies, and nucleic acids) on the surface of particles.^[^ ^75^ ^]^ Nowadays, numerous ligand‐modified nanoparticles have been developed to modulate the protein‐protein, protein‐cell, and cell‐cell interactions.^[^ ^108^, ^109^ ^]^ For example, Our group demonstrated an Aβ recognition element‐modified nanoparticle, which could change the morphology of Aβ aggregates, resulting in the formation of Aβ/nanoparticle co‐assembled nanoclusters instead of Aβ oligomers. With the reduction of the pathological Aβ oligomers, this nanoparticle attenuated Aβ‐induced neuron apoptosis.^[^ ^110^ ^]^ Recently, our group prepared an antibody‐like polymeric nanoparticle (denoted as APN) modified with galactose and Tuftsin peptides, which could specifically capture immunosuppressive galectin‐1 and activate macrophage‐mediated phagocytosis in the tumors. As a result, APN facilitated the removal of galectin‐1 by macrophage in tumor tissues, thereby improving the antitumor T‐cell responses.^[^ ^111^ ^]^ Kim et al. used carboxylated polystyrene nanoparticle as the substrate and created a multivalent bi‐specific nano‐bioconjugate engager (denoted as mBiNE), which could promote immune‐mediated eradication of tumor cells. By simultaneously immobilizing anti‐HER2 antibodies and calreticulin (CRT, a phagocytic signal) onto the carboxylated polystyrene nanoparticle, mBiNE stimulated HER2‐targeted phagocytosis and induced robust and durable antitumor T‐cell responses against HER2^+^ tumors.^[^ ^109^, ^112^ ^]^ These studies demonstrated the capability of polymeric nanoparticles as a promising platform to modulate protein‐protein, protein‐cell, and cell‐cell interactions.

#### 2.2.3 Carbon‐based nanomaterials

Over the past decades, carbon‐based nanomaterials have gained increasing interest and been investigated for biomedical applications. Among these nanomaterials, carbon nanotubes, graphene, and graphene oxide have received considerable attentions due to their unique structural and mechanical properties.^[^ ^113^ ^]^ Nowadays, carbon nanotubes and graphene have been widely used for tissue engineering.^[^ ^114^ ^]^ Carbon nanotubes are molecular‐scale hallow tubes consisting of carbon atoms, which have robust mechanical strength and high flexibility. For example, Tan et al. found that carboxylated multi‐walled carbon nanotubes (MWCNTs) were able to promote cell adhesion and neurogenic differentiation of mesenchymal stem cells without inducing factors, but had no effect on osteogenic differentiation.^[^ ^115^, ^116^ ^]^ On the contrary, Watari et al. reported that the films of MWCNTs could effectively induce osteogenic differentiation of mesenchymal stem cells in the absence of differentiation inducing agents.^[^ ^116^ ^]^ Graphene is a one‐atom‐thick membrane of carbon atoms arranged in the honeycomb lattice. Compared with carbon nanotubes, graphene presents an open surface and high specific surface area for chemical modification of ligands or non‐covalent interaction with biomolecules. More importantly, graphene has a high Young's modulus (E, 0.5–1 TPa) than other materials, whereas it is not brittle.^[^ ^117^ ^]^ Therefore, graphene is an ideal alternative for tissue engineering, especially the bone regeneration. Graphene oxide is a highly oxidized form of graphene through oxidation of graphite, which has also gained interests in regenerative medicine or other biomedical applications.^[^ ^118^ ^]^ For example, Bai et al. demonstrated a biomimetic hydroxyapatite mineralization induced by poly‐dopamine‐functionalized reduced graphene oxide (denoted as RGO‐PDA). MC3T3‐E1 cells on RGO‐PDA substrates showed higher cellular activities including adhesion, proliferation, and osteogenic differentiation than the bare glass substrates.^[^ ^119^ ^]^ Yang et al. found that the graphene oxide could effectively activate the autophagy of neurons and microglial cells both in vitro and in vivo by inhibiting the mTOR signaling pathway.^[^ ^120^ ^]^ The activation of autophagy significantly improved the phagocytosis capacity of neurons and microglial cells, which showed great potentials of removing β‐amyloid (Aβ) protein in the brain for neuroprotection.^[^ ^121^ ^]^

In addition, similar to the metal‐based nanomaterials, carbon nanomaterials including carbon nanotubes and graphene oxide are also found to exhibit catalytic activities.^[^ ^83^ ^]^ Nanozymes constructed by carbon nanomaterials has demonstrated lower manufacturing cost and higher catalytic stability than several natural enzymes. Although carbon materials have a high academic and clinical importance, and are considered inert to cells and tissues, it should be noted that their reactivity increases drastically at the nanoscale. Thus, there is a demand for assessments of the potential toxicity of carbon‐based nanomaterials in future studies.

#### 2.2.4 Supramolecular‐based nanomaterials

Supramolecular chemistry is closely linked to life science.^[^ ^122^, ^123^ ^]^ Self‐assembly and molecular recognition, which play critical roles in living organisms, have inspired the development of supramolecular‐based nanomaterials with unique architectures and properties.^[^ ^124^ ^]^ Compared with covalent bonding, the noncovalent bonding present in supramolecular‐based nanomaterials is dynamic and reversible, which displays a series of advantages. For example, supramolecular‐based nanomaterials are formed by the interactions of different supramolecular monomers through noncovalent interactions (e.g., hydrogen bonds, hydrophobic interactions, electrostatic interactions, and π‐π stacking forces), thereby avoiding tedious preparation and purification procedures.^[^ ^125^ ^]^ Moreover, the dynamic property and adaptive behavior of noncovalent bonding allow for the convenient dissociation and reconstruction of supramolecular‐based nanomaterials both in vitro and in vivo. Finally, the design of supramolecular‐based nanomaterials uses the “bottom‐up” principle, thereby providing a reliable approach to control the size and morphology of the materials.^[^ ^126^ ^]^

Among all the supramolecular monomers reported, peptides are the most attractive building blocks for constructing bioactive materials due to their bioactivity, biodegradability, and biocompatibility.^[^ ^127^ ^]^ More importantly, peptides with specific sequences can be conveniently synthesized to possess both hydrophilic and hydrophobic domains in one monomer.^[^ ^128^ ^]^ With these properties, they have been widely used in storing bioinformation, disease diagnosis, tissue engineering, and drug delivery.^[^ ^129^ ^]^ Lam et al. reported an “in vivo self‐assembly” strategy for in situ construction of bioactive nanomaterials. In this study, they prepared a transformable peptide monomer that could self‐assemble into nanoparticles, however, the particles transformed into nanofibers when binding to the HER2 on the surface of tumor cells. As a result, the nanofibers disrupted dimerization of HER2 and subsequent downstream signal, thereby killing the tumor cells in mouse xenograft models.^[^ ^55^ ^]^ Recently, researchers from the same group also demonstrated the capability of transformable peptides to inhibit bacterial invasion in vivo by mimicking the mechanisms of antimicrobial peptide human defensin‐6. The transformable peptides consisted of a self‐assembling peptide sequence and a ligand peptide sequence. In this design, the transformable peptides could recognize bacteria via ligand‐receptor interactions and trap the bacteria through in situ formation of nanofibrous networks.^[^ ^130^ ^]^ In addition, “in vivo self‐assembly” strategy has also been used as a promising drug delivery system to enhance the drug accumulation in tumor tissues. For example, Yu et al. developed oxidation‐regulated self‐assembly of peptides as drug carriers for cancer therapy. The peptides underwent efficient oxidation‐regulated self‐assembly to form nanofibers for enhanced tumor accumulation.^[^ ^17^, ^39^ ^]^ The oxidation of methionine of the peptide in reactive oxygen species (ROS)‐rich tumor tissue promoted the morphology transformation. Moreover, co‐assembling the peptides by using their derivatives modified with chemotherapeutic agents or photosensitizer conferred therapeutic ability to these nanofibrils.

Besides peptides, macrocyclic amphiphiles such as cyclodextrins and calixarenes are also emerging monomers for the construction of supramolecular‐based nanomaterials, which have been used to construct bioactive surface for molecular recognition.^[^ ^126^, ^131^ ^]^ For example, Guo et al. co‐assembled the cyclodextrins and calixarenes into one assembly to construct a bioactive surface with a high affinity and selectivity to the Aβ protein. This assembly with unique surface could effectively inhibit Aβ fibrillation via host‐guest recognition, thereby attenuating Aβ‐induced neuron damages.^[^ ^132^, ^133^, ^134^ ^]^ Recently, Guo et al. reported another assembly prepared by macrocyclic amphiphiles which could accurately capture the macromolecular toxins such as melittin in the blood. As a result, this assembly inhibited the interactions of melittin with cell membranes, alleviated melittin cytotoxicity and hemolytic toxicity, eventually improved the survival rate of melittin‐poisoned mice.^[^ ^135^ ^]^ It is important to point out that, although supramolecular‐based nanomaterials have been reported for a long time, exploring their bioactivities is still an emerging research area. We believe that supramolecular‐based nanomaterials would bring new development opportunities for bioactive nanomaterials.

## 3 BIOMEDICAL APPLICATIONS OF BIOACTIVE NANOMATERIALS

In the past few decades, various types of bioactive nanomaterials have been developed. In this section, we summarize several typical biomedical applications of bioactive nanomaterials, including wound healing, cancer immunotherapy, neurodegenerative disease therapy, and biocatalyst.

### 3.1 Wound healing

Wound healing is a forced response to internal or external stimulus that damage tissues or any organs.^[^ ^157^ ^]^ In the case of tissue injury, our body undergoes a series of spontaneous self‐repairing processes, including hemostasis, anti‐infections, cell proliferation, tissue remodeling, and eventually restores the injured tissues.^[^ ^158^ ^]^ The time span of healing process largely depends on the extent of tissue damages. In more severe injuries, the body cannot adequately repair itself, thus falling into chronic and non‐healing wounds.^[^ ^157^, ^159^ ^]^ Various bioactive nanomaterials have been developed or under development to promote/assist wound healing due to their biocompatibility, antimicrobial effects, and drug loading capabilities. In this section, we will discuss the recent advances in the development of bioactive wound healing nanomaterials in promoting hemostasis, anti‐infection, and proliferation.

#### 3.1.1 Hemostasis

**FIGURE 2:**
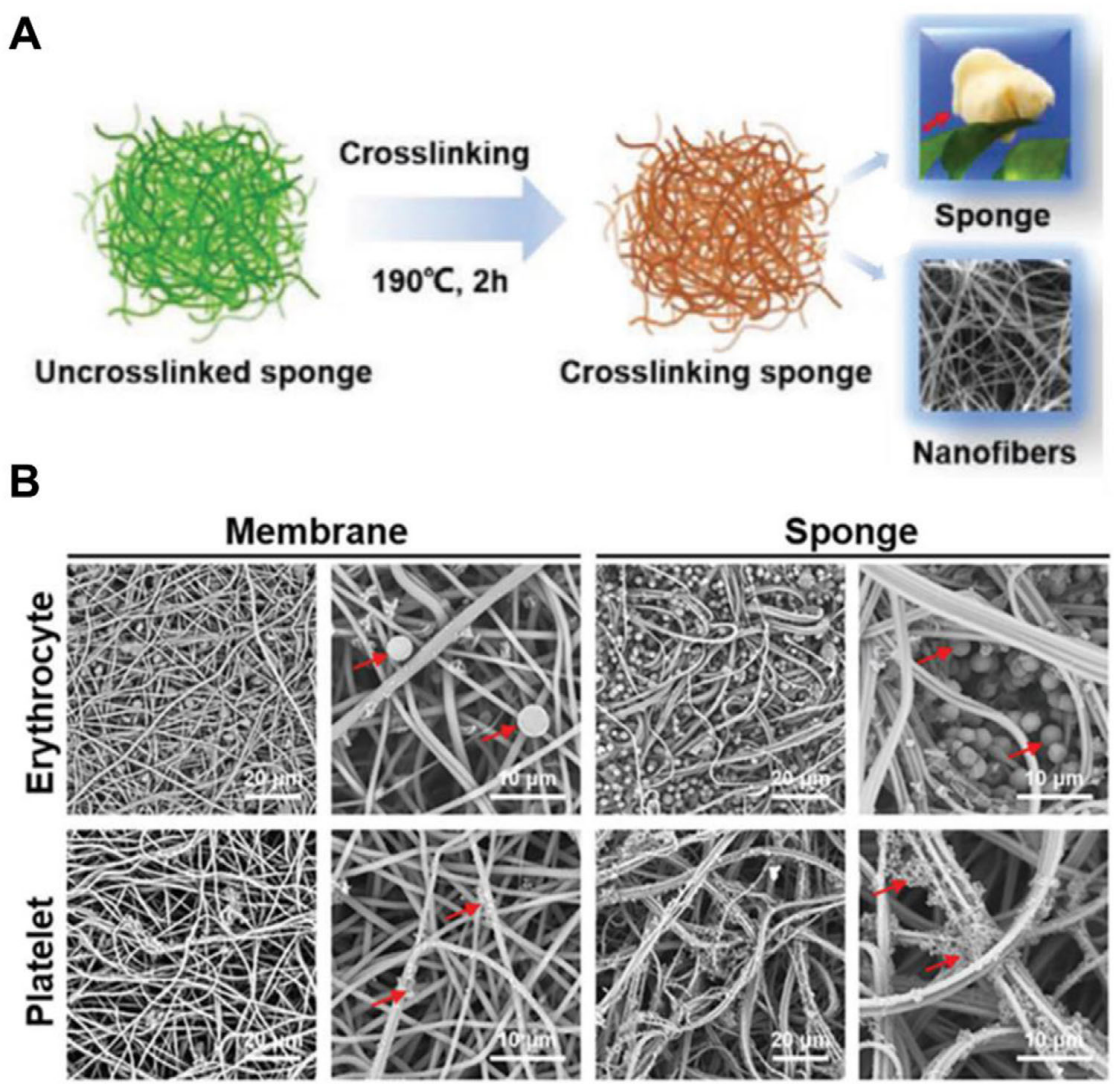
Gelatin‐based nanofiber sponge for rapid hemostasis. (A) Schematic illustration of 3D nanofiber gelatin sponge for efficient hemostasis. (B) SEM images of erythrocytes and platelets adhesion on gelatin nanofiber membrane and sponge. Adapted with permission.^[^ ^162^ ^]^ Copyright 2021, John Wiley & Sons

Hemostasis is the first and crucial stage of wound healing, aiming to seal the injury site or ruptured blood vessel.^[^ ^160^ ^]^ In most operations, traditional materials such as gauze or cotton wool are employed as dressing materials to stop bleeding and cover injured site, but the hemostasis effect of these conservative strategies are usually unsatisfactory.^[^ ^161^ ^]^ The development of nanotechnology provided new opportunities for effective hemostasis. For example, nanomaterials can be constructed with a larger surface area and porous structure, which are able to adsorb blood and stop bleeding rapidly. Tian et al. presented a rapid hemostatic sponge by co‐assembling mesoporous silica nanoparticles (MSNs) with glycerol‐modified *N*‐alkylated chitosan (GACs).^[^ ^136^ ^]^ The as‐prepared MSN‐GACs exhibited significant higher blood adsorptions and hemostatic ability than commercial Combat Gauze (CG). Mo et al. presented a gelatin‐based nanofiber sponge for rapid hemostasis (Figure 2).^[^ ^162^ ^]^ The nanofibers were used to construct a macroporous structure, which allows for the rapid liquid diffusion and ultrastrong water absorption. Similarly, cross‐linked graphene with hierarchical porous structures was also developed as hemostatic material. In addition, nanomaterials can be easily sprayed or injected onto injury sites to in situ form nanofiber or hydrogel to effectively stop bleeding. Recently, self‐assembly peptides (SAPs),^[^ ^154^, ^163^, ^164^ ^]^ cyanoacrylate (CA),^[^ ^165^, ^166^ ^]^ nano‐chitosan,^[^ ^167^, ^168^ ^]^ alginate,^[^ ^169^ ^]^ and MSN‐integrated nanomaterials^[^ ^170^ ^]^ have shown their efficacy in hemostasis. Among all these bioactive nanomaterials, peptides are the most attractive building blocks to construct nanofiber and hydrogel due to their biodegradability and biocompatibility. More importantly, the structure and property of these assemblies can be easily tailored by changing the sequence of peptides. In 2006, Behnke et al. reported the first applications of SAPs in hemostasis.^[^ ^154^ ^]^ They discovered a novel peptide, RADA16 (AcN‐RADARADA‐RADARADA‐CONH_2_) that quickly assembled into nanofibers and form a jelly‐like hydrogel after spraying onto or injecting into the injury site, and stopped bleeding within 10–15 s. In the following ten years, a series of SAPs, such as IAP (Ac‐KLLKLLLKLLKLLLKLLLKLLK‐CONH_2_),^[^ ^171^ ^]^ KOD ((PLG)_4_(PHG)_4_(AHG)_4_),^[^ ^172^ ^]^ RATEA16 (CH_3_CO‐RATARAEARATARAEA‐CONH_2_),^[^ ^173^ ^]^ SPG‐178 (RLDLRLALRLDLR),^[^ ^174^ ^]^ etc. were developed, which have greatly improved the quality of patient's life. Moreover, the hemostatic effect of SAPs can be further improved by integrating functional fragments into the peptides. Wang et al. conjugated two ECM‐related motifs GRGDS (a ligand that can specifically bind to fibronectin receptor) and YIGSR (extracted from laminin‐1) onto RADA16 to form RADA16‐GRGDS and RADA16‐YIGSR, respectively.^[^ ^164^ ^]^ The resultant nanofibers and hydrogels exhibited significant higher binding affinities to ECM and could completely stop bleeding in a few seconds. However, the inherent disadvantage of peptides is expensive, which greatly limited the general applications of peptide‐based hemostatics. Development of novel peptide synthesis method to reduce the cost of peptide is essential. In addition, exploring novel nanomaterials to replace peptides is also a promising approach for hemostasis.

#### 3.1.2 Anti‐wound infections

**FIGURE 3:**
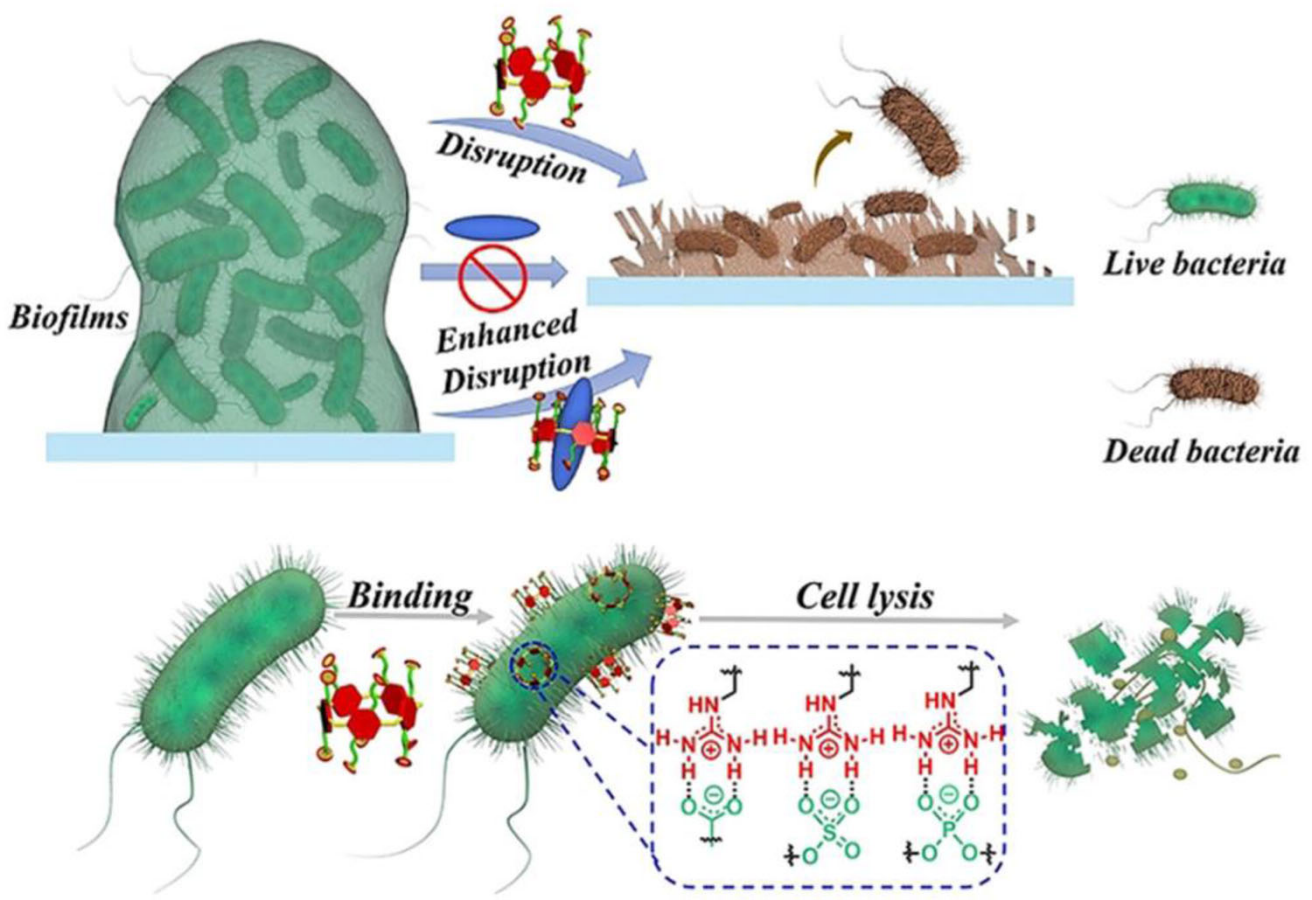
Schematic illustration of guanidinium‐modified pillar[5]arene (GP5) for antimicrobial applications. The GP5 can quickly bind with the anionic components on biofilms and the phospholipids of material membrane via salt bridges, thereby destroying the membrane integrity and finally causing cell lysis. Adapted with permission.^[^ ^155^ ^]^ Copyright 2021, John Wiley & Sons

**FIGURE 4:**
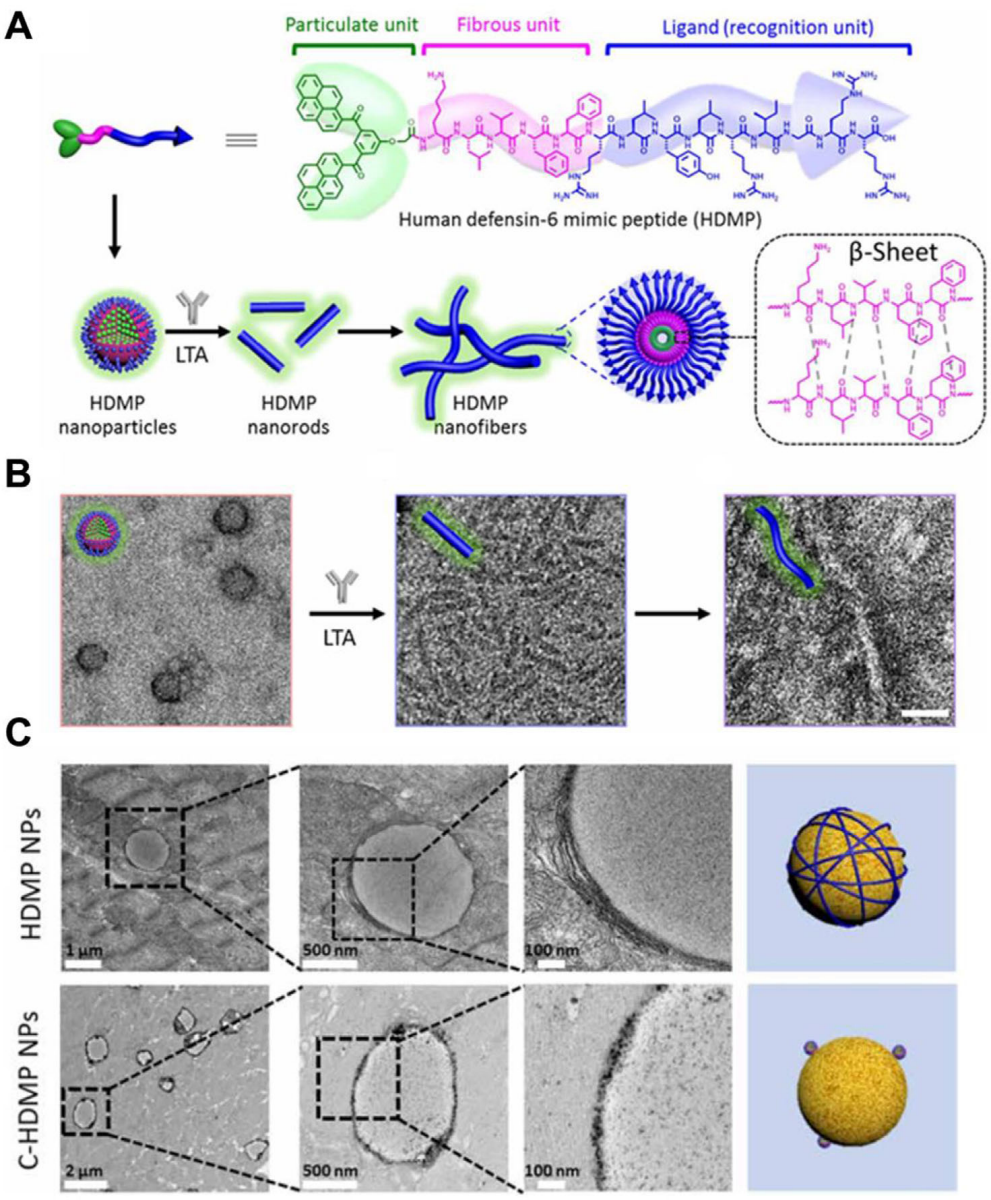
HDMP for antibacterial applications. (A) Molecular structure of HDMP and schematic illustration of HDMP assembly into NPs, transforming into nanorods and nanofibers. (B) TEM images of HDMP nanoparticle, HDMP nanorods, and HDMP nanofibers. (C) TEM images of muscle tissue slices, showing transformed HDMP nanofibers and maintained C‐HDMP NPs on bacterial surfaces. Adapted with permission.^[^ ^130^ ^]^ Copyright 2020, AAAS

Wound infection is a major cause of impaired wound healing. In the injured area, the skin barrier is greatly damaged, which cannot effectively prevent the invasion of external microorganisms.^[^ ^175^ ^]^ The infection can impede wound healing and, in severe cases, may even cause amputation or death.^[^ ^176^ ^]^ Therefore, anti‐wound infection is essential for wound healing after hemostasis. To date, a variety of bioactive nanomaterials including metal‐based nanocomposites,^[^ ^177^ ^]^ graphene‐based nanomaterials,^[^ ^178^ ^]^ and polymer‐based NPs^[^ ^179^, ^180^ ^]^ have shown their efficacy in antimicrobial. These nanomaterials have different antimicrobial mechanism and can be roughly summarized into three categories. (1) Nanomaterials that contain guanidinium (GUA) and quaternary ammonium (QA) groups can interact strongly with cell membrane, disrupt the integrity of biofilms, and trigger the release of intracellular contents. The binding affinities of these materials to cell membrane is positively correlated to the length of alkyl chain on GUA/QA groups, with maximum affinities at the chain length of 16 (QA)^[^ ^181^, ^182^ ^]^ and 4 (GUA).^[^ ^179^ ^]^ In addition to adjusting the length of alkyl chain, this affinity can also be improved by integrating GUA and QA into the macrocyclic molecule. For example, Cohen et al. constructed a series of quaternary phosphonium (QP) and QA‐decorated pillar[5]arenes (with same alkyl chain length of 3) for antimicrobial applications (Figure 3).^[^ ^183^ ^]^ Similarly, Wang et al. constructed a novel GUA‐modified pillar[5]arene (GP5).^[^ ^155^ ^]^ These pillar[5]arene analogs can quickly bind with the anionic components on biofilms and the phospholipids in bacteria membrane via salt bridges, thereby destroying the membrane integrity and finally cause bacterial lysis. (2) Nanomaterials that can trap or arrest bacteria to inhibit the bacterial invasion. For example, Wang et al. presented a human defensin‐6 mimic peptide (HDMP) that can specifically recognize bacteria and in situ form nanofibrous networks to trap bacteria (Figure 4).^[^ ^130^ ^]^ The trapped bacteria are unable to proliferate or invade host cells, thereby significantly improving the survival rate of infected mice. Similarly, Qiao et al. developed a novel dopamine‐modified MSN (DOPA/MSN) for effective wound healing.^[^ ^137^ ^]^ In addition to adsorbing blood and stopping bleeding, DOPA/MSN self‐polymerized onto bacterial, hindered the absorption of nutrition and the release of metabolic waste, and ultimately caused bacterial lysis. (3) Nanomaterials that can generate ROS to directly kill bacteria. ROS, especially singlet oxygen, once produced in large quantities, will immediately induce intracellular oxidation, change membrane potential and release intracellular contents, thus leading to bacteria death. Various metal‐based nanocomposites^[^ ^184^ ^]^ (ZnO, TiO_2_, MgO, V_2_O_5_, AgNPs) and graphene‐based nanomaterials have shown their effectiveness in generating ROS and killing bacteria without developing antibiotic resistance. However, these metal‐based nanocomposites are usually unstable in solution and are prone to aggregate, thus significantly reducing their antibacterial efficacy. Therefore, these nanoparticles are often modified with surfactant such as tea polyphenol (TP), β‐cyclodextrin, chitosan, polyvinylpyrrolidone (PVP), zwitterionic, etc. before applications.^[^ ^185^ ^]^

#### 3.1.3 Promote proliferation

Generally, the proliferative phase begins within days after injury and lasts for about 14 days. In this process, endothelial cells and fibroblasts proliferate rapidly and participate in the formation of new capillaries (angiogenesis) and new ECM.^[^ ^186^ ^]^ In addition, myofibroblasts also migrate into the wound rim from the surrounding tissue to contracting the wound area.^[^ ^187^, ^188^ ^]^ Recent studies have revealed that several bioactive nanomaterials including metal‐based nanocomposites (AgNPs, AuNP, nanoceria, etc.),^[^ ^189^ ^]^ bioglass,^[^ ^190^ ^]^ carbon‐based nanomaterials (nanotubes, graphene),^[^ ^191^, ^192^ ^]^ nitric oxide‐carrier,^[^ ^193^ ^]^ etc. can effectively promote cell proliferation. These nanomaterials share similar biological mechanisms, mainly achieved by upregulating growth factors‐related genes such as VEGF, endothelial growth factor receptors (EGFR), or fibroblast growth factor 2 (FGF2). However, the chemical mechanisms of these materials remain unclear and have seldom been investigated. Future efforts should focus on exploring their chemical mechanisms. In addition, nanomaterials can act as scaffold to promote fibroblast migration. For example, Zhang et al. presented graphene‐based nanocomposites (_A_CG NCs)^[^ ^194^ ^]^; Kristl et al. constructed a poly(vinyl alcohol) (PVA)‐based nanofiber^[^ ^195^ ^]^; Zhou et al. developed a halloysite nanotube (HNT).^[^ ^196^ ^]^ These nanomaterials can effectively interact with fibroblasts to promote their migration and ultimately accelerate wound healing.

### 3.2 Immune modulation for cancer immunotherapy

Cancer immunotherapy revolutionarily mobilizes the host's immune system to recognize and eliminate tumor cells, has greatly shifted the paradigm of cancer treatment.^[^ ^197^ ^]^ Compared with traditional treatments, cancer immunotherapy has a durable antitumor effect with reduced metastasis and recurrence.^[^ ^198^ ^]^ However, more than 70 % of patients are only transiently or not responsive to immunotherapy,^[^ ^199^ ^]^ mainly due to (1) insufficient activation of immune system and (2) the immunosuppression of the tumor microenvironment (TME). In the past few decades, nanomaterials have been widely used as delivery vectors to improve the bioavailability of immune modules for cancer immunotherapy. In addition to their delivery functions, more and more studies have shown that certain types of nanomaterials can inherently regulate the immune system. In this section, we will discuss the recent advances in the development of bioactive immune modulation nanomaterials in activating immunity and alleviating immunosuppressive TME.

#### 3.2.1 Activate T cell‐mediated immunity

Current research in cancer immunotherapy is mainly based on T cell‐mediated cellular immunity. The effective activation of T‐cell mediated antitumor immunity requires a series of stepwise events, namely “cancer‐immunity cycle.”^[^ ^200^ ^]^ However, in most cases, this cycle may be blocked at one or more steps, resulting in insufficient activation of immune system. Recent studies indicate that nanomaterials can participate in the certain steps of cancer‐immunity cycle, such as promoting antigen presentation and APC maturation, thus helping to initiate or reinitiate a self‐sustaining immune cycle.

##### Promote antigen presentation

**FIGURE 5:**
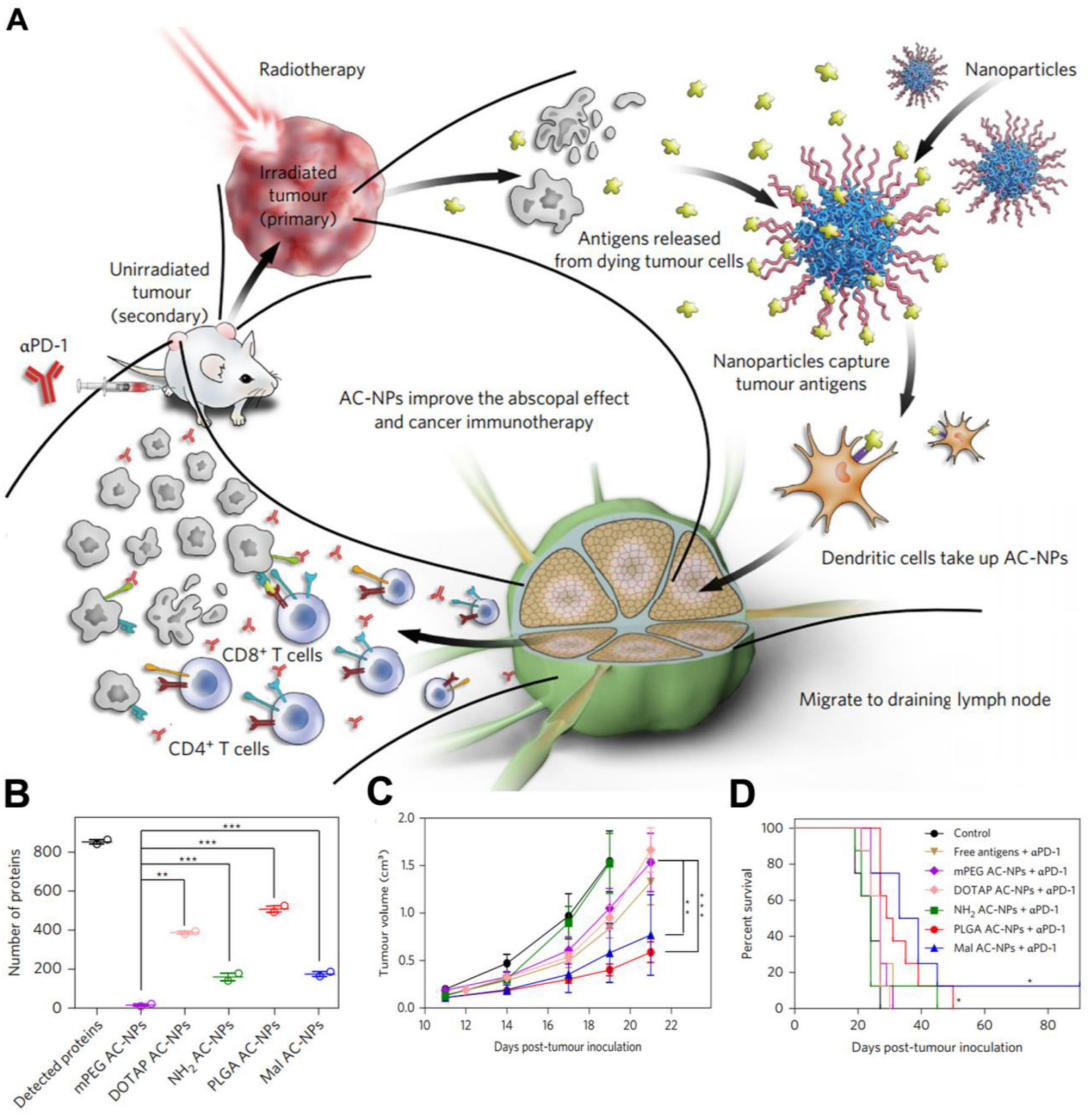
AC‐NPs promote antigen presentation for efficient cancer immunotherapy. (A) Schematic depiction of AC‐NPs to capture antigen and promote antigen presentation. (B) The amount of proteins captured by AC‐NPs. (C) Tumor size in mice after receiving different AC‐NPs. (D) Survival curves of the mice after receiving different AC‐NPs. Adapted with permission.^[^ ^72^ ^]^ Copyright 2017, Nature Publishing Group

Bioactive nanomaterials can regulate the interactions between the tumor‐derived protein antigens (TDPAs) and APCs, thereby enhancing the uptake and presentation of tumor antigens by APCs. For these nanomaterials, the surface property is a critical parameter. According to their surface properties, bioactive nanomaterials can be summarized into three categories: (1) the surface is hydrophobic, (2) the surface is positively charged, and (3) the surface contains reactive groups. Bioactive nanomaterials with hydrophobic surface can capture TDPAs through hydrophobic–hydrophobic interactions. For example, Wang et al. constructed a PLGA‐based biodegradable antigen capture nanoparticle (AC‐NPs) that can effectively capture and delivery TDPAs to APCs (Figure 5).^[^ ^72^ ^]^ With this strategy, the AC‐NPs significantly promoted the presentation of antigens to T cells and effectively activated T‐cell mediated antitumor immune response. Moreover, the AC‐NPs improved the efficacy of ICB‐based immunotherapy in B16F10 melanoma‐bearing mice, which generated up to a 20% survival rate compared with the mice receiving ICB monotherapy. Bioactive nanomaterials with positive‐charged surface can capture TDPAs through ionic interactions. Hu et al. presented a mannose modified stearic acid‐grafted chitosan micelle (MChSA) for personalized immunotherapy.^[^ ^201^ ^]^ MChSA can effectively capture endogenous antigens and target tumor‐draining lymph node. As a result, MChSA significantly promoted antigen presentation, induced robust T cell responses, and ultimately inhibited tumor growth. Bioactive nanomaterials with reactive groups‐containing surface can capture TDPAs through chemical reaction. For example, Chen et al. presented an upconversion nanoparticle (UCNP) based antigen‐capturing nanoplatform (UCNP/ICG/RB‐mal) for metastatic cancer treatment.^[^ ^139^ ^]^ Upon irradiation, UCNP/ICG/RB‐mal (maleimide, mal) can effectively capture TDPAs by reacting with the exposed sulfhydryl (‐SH). The captured TDPAs are then delivered to APCs, thereby promoting antigen presentation and inducing tumor‐specific immune responses.

##### Promote APC maturation

**FIGURE 6:**
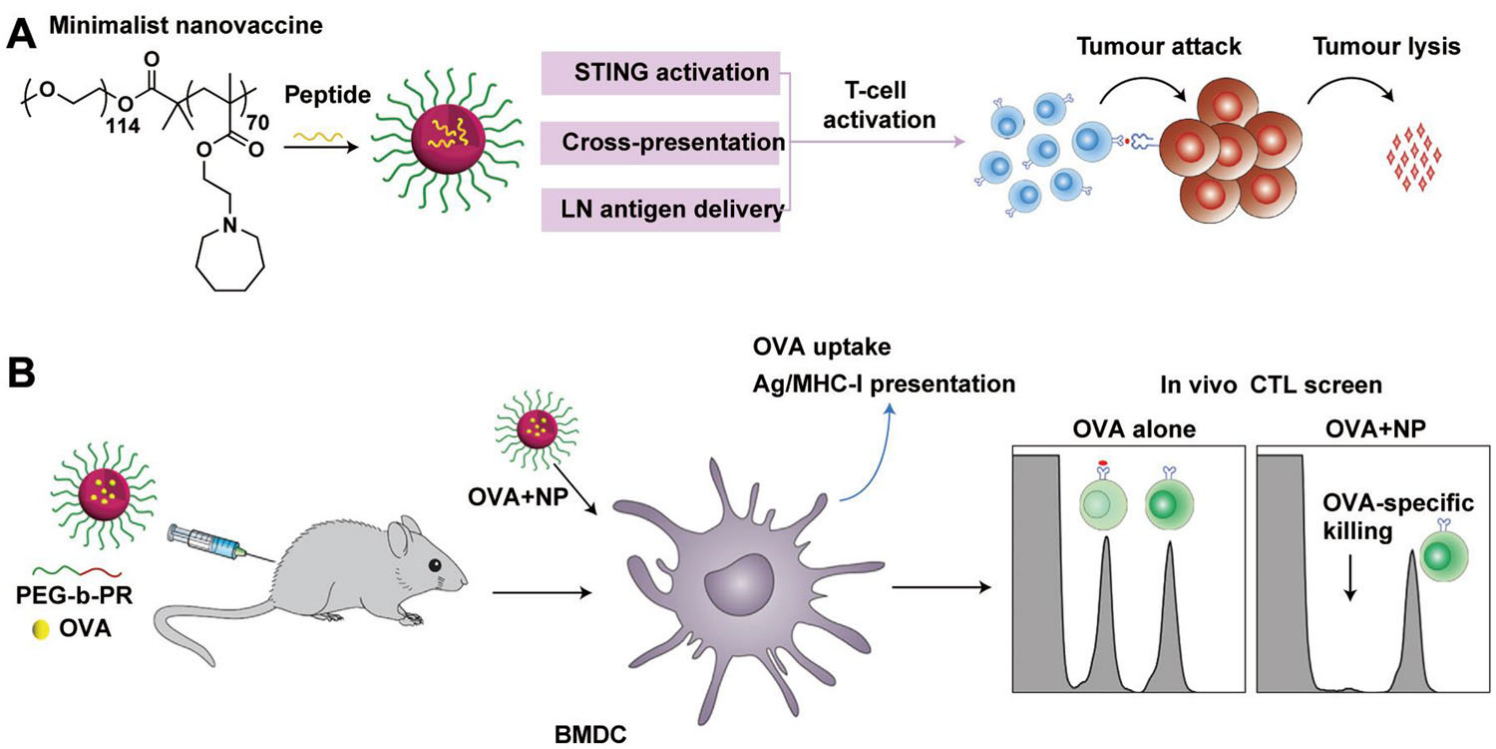
Schematic representation of the PC7A‐NPs for efficient cancer immunotherapy. (A) Schematic for the design of PC7A nano‐vaccine and the potential mechanism to activate antitumor immune response. (B) Schematic for PC7A‐NPs to generate a strong OVA‐specific cytotoxic T lymphocyte response. Adapted with permission.^[^ ^148^ ^]^ Copyright 2017, Nature Publishing Group

Bioactive nanomaterials can initiate inflammatory responses to promote APC maturation. Generally, this inflammatory response was initiated by promoting the secretion of inflammatory cytokines. Gao et al. reported a polymeric nanoparticle (PC7A‐NP)‐based nanovaccine that can stimulate the secretion of interferon for efficient cancer immunotherapy (Figure 6).^[^ ^148^ ^]^ After subcutaneous injection, PC7A‐NP effectively transport to lymph node and activate the stimulator of interferon genes (STING) pathway. With this strategy, PC7A‐NP promotes the maturation of APC and effectively activates T‐cell based antitumor immune response. As combined with anti‐PD‐1 antibody, PC7A‐NP demonstrated great tumor suppression, with almost 100% survival within observed 60 days. In addition, activating the complement system can also promote the release of inflammatory cytokines. Reddy et al. reported a pluronic‐stabilized polypropylene sulfide (PPS) nanoparticles that can activate the complement system in a hydroxyl (‐OH)‐dependent manner.^[^ ^202^ ^]^ They also found that the activating efficiency of complement system is related to the surface charge of nanomaterials, in which lower surface charge can induce a higher activation.^[^ ^203^ ^]^ Besides the secretion of inflammatory cytokines, bioactive nanomaterials can also directly activate inflammatory cytokines receptors to promote APC maturation. Akashi et al. reported an amphiphilic poly(amino acid) nanoparticle (γ‐PGA–Phe NPs) that can directly activate toll‐like receptors 4 (TLRs).^[^ ^204^, ^205^ ^]^ Dwivedi et al. reported a zinc oxide nanoparticles (ZNPs) that can activate TLRs 6 pathway.^[^ ^206^ ^]^ By directly activating inflammatory cytokines receptors, these nanoparticles effectively promote the maturation of DCs and induce robust T‐cell mediated antitumor immunity.

#### 3.2.2 Alleviate immunosuppressive TME

**FIGURE 7:**
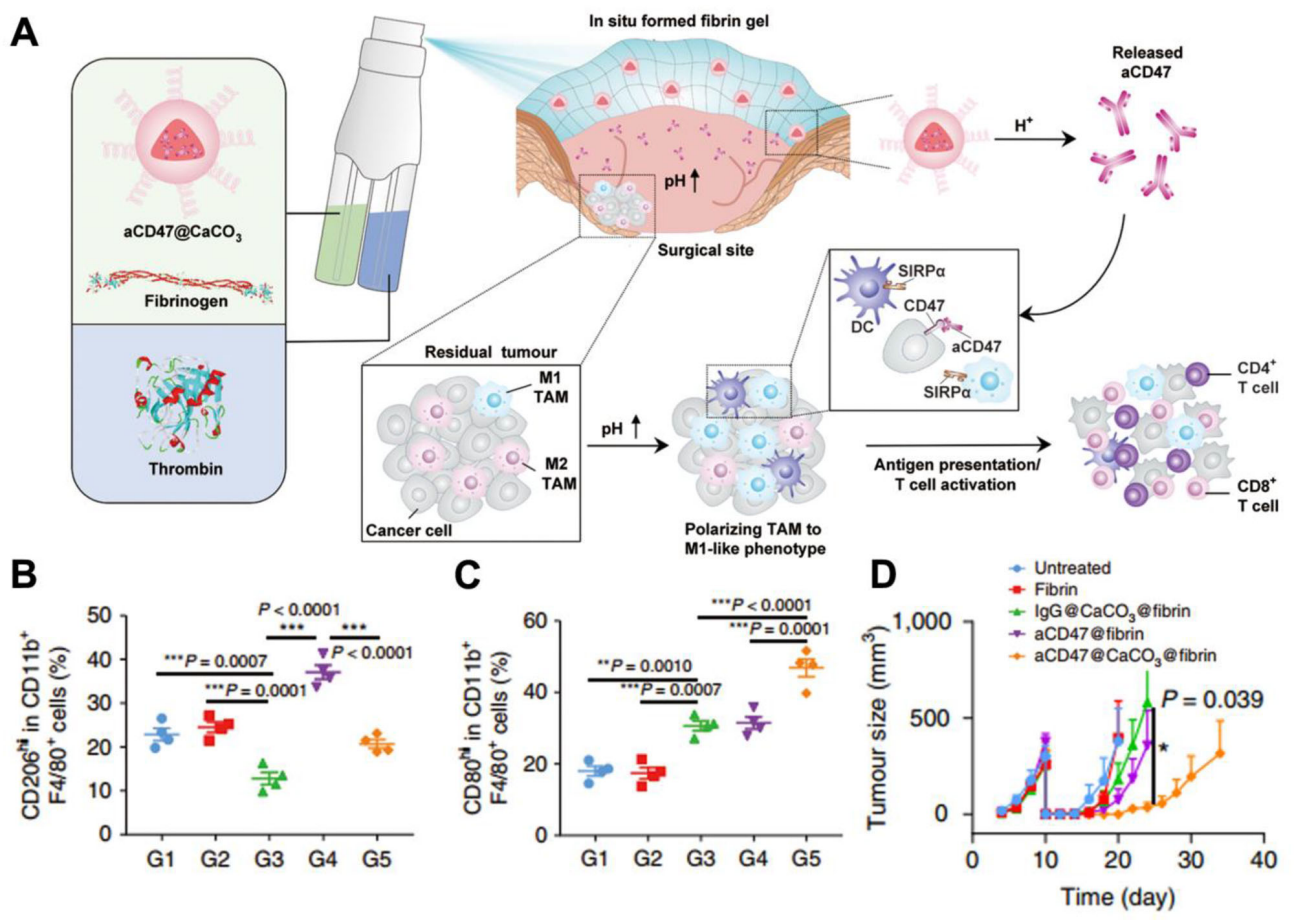
aCD47@CaCO_3_ repolarizing the phenotype of TAM for post‐surgical treatment. (A) Schematic of aCD47@CaCO_3_ nanoparticles to scavenge the H^+^ and repolarize the phenotype of TAM within the post‐surgery tumor bed. Quantitative analysis of (B) M2‐like macrophages and (C) M1‐like macrophages after different treatments. (D) Tumor size in mice after receiving different treatments. Adapted with permission.^[^ ^140^ ^]^ Copyright 2019, Nature Publishing Group

The immunosuppressive TME is another factor that restricts the efficacy of cancer immunotherapy. TME is a heterogeneous environment composed of a series of inflammatory cytokines/chemokines and immunosuppressive cells.^[^ ^207^ ^]^ Bioactive nanomaterials can effectively relieve immunosuppressive TME through depleting or re‐polarizing immunosuppressive cells. Tumor‐associated macrophages (TAM) is an important fraction of infiltrating immune cells in tumor and are typically polarized into M2‐like TAMs (exert pro‐tumorigenic activities).^[^ ^208^ ^]^ Gu et al. developed an anti‐CD47 antibody loaded calcium carbonate nanoparticles (aCD47@CaCO_3_) for post‐surgical cancer treatment (Figure 7).^[^ ^140^ ^]^ CaCO_3_ can scavenge the H^+^ in TME to repolarize the phenotype of TAM from M2‐like TAMs to M1‐like TAMs (exert antigen presentation activities). The locally released aCD47 can effectively block the “don't eat me” signal on tumors, thus cooperating effectively to regulate the immunosuppressive TME. Kim reported a folate‐functionalized bioactive glass nanoparticle BGN(F).^[^ ^138^ ^]^ The folate on BGN(F) can effectively target M2‐like TAMs and release Si^4−^/Ca^2+^ ions to repolarize the phenotype of TAM. In addition, glycocalyx‐mimicking nanoparticles,^[^ ^209^ ^]^ mannosylated liposome,^[^ ^210^ ^]^ ferumoxytol,^[^ ^211^ ^]^ and several polymeric nanoparticles^[^ ^212^ ^]^ also show their potentials in repolarizing TAMs.

**FIGURE 8:**
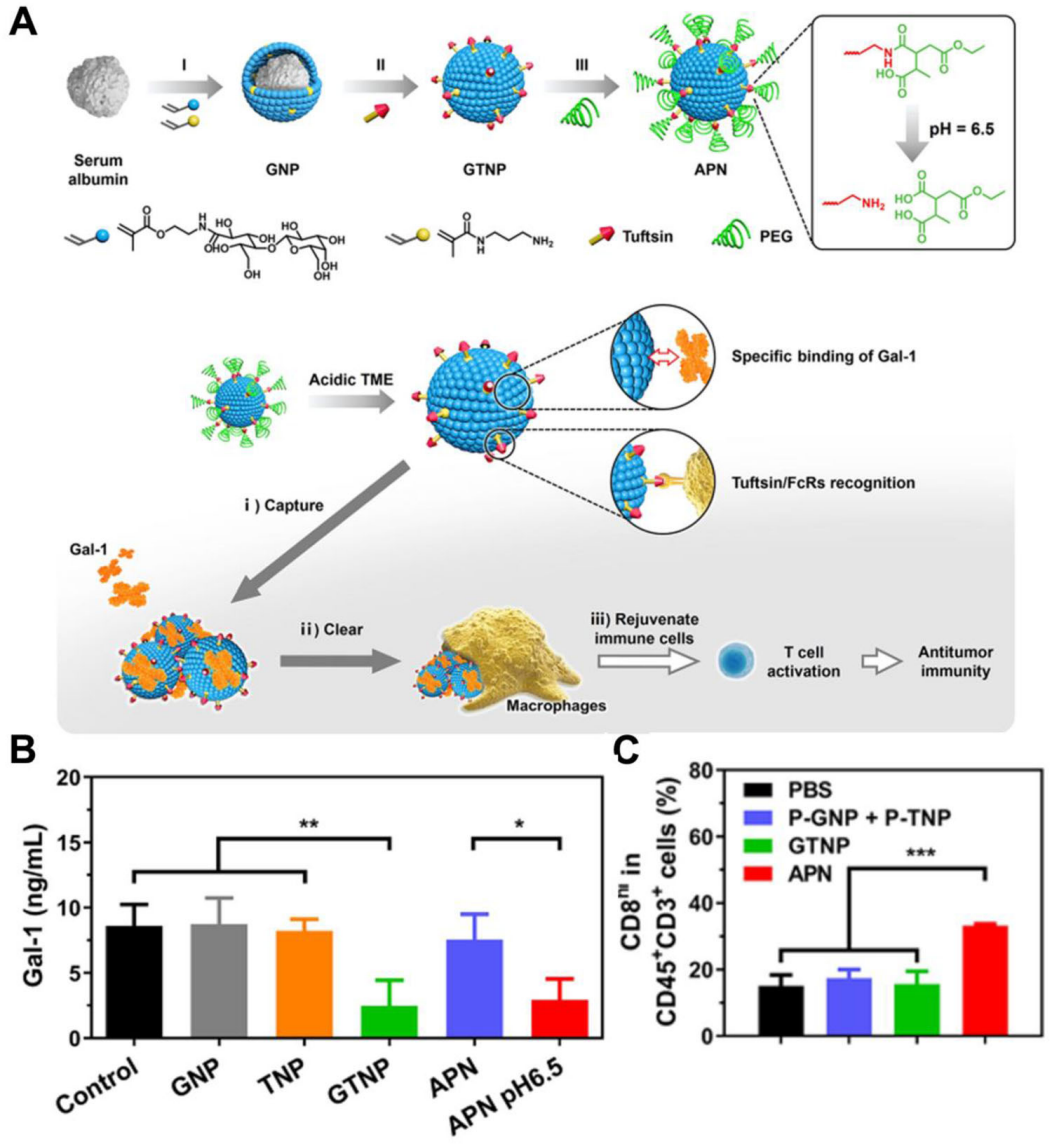
APN for the effective removal of intratumoral galectin‐1. (A) Schematic for the preparation of APN and its mechanism in facilitating macrophages‐mediated removal of intratumoral Gal‐1. (B) Elisa determination of Gal‐1 in culture medium after incubated with GNP, TNP, GTNP, and APN. (C) Quantitative analysis of CD8^+^ T after different treatments. Adapted with permission.^[^ ^111^ ^]^ Copyright 2021, American Chemical Society

Removal of immunosuppressive factors can also relieve the immunosuppressive TME. Our group recently reported an antibody‐like polymeric nanoparticle (APN) for the effective removal of intratumoral galectin‐1 (Gal‐1, an immunosuppressive factor) (Figure 8).^[^ ^111^ ^]^ The surface of APN was a cross‐linked polymer layer that contains two types of functional groups, Tufstin peptides (TKPR) and galactose units, in which the galactose can bind to Gal‐1 and Tufstin can activate macrophage‐mediated phagocytosis.^[^ ^213^ ^]^ Therefore, APN function like a linker between Gal‐1 and macrophages to promote macrophage‐mediated Gal‐1 clearance. With this strategy, the APN effectively relieves immunosuppressive TME and elicit robust antitumor immune response. As far as we know, APN is the first bioactive material reported so far that can eliminate immunosuppressive factors, which provides new ideas for the design of novel bioactive materials to eliminate other factors or toxins.

### 3.3 Neurodegenerative disease therapy

Neurodegenerative disease is a type of disease caused by the gradual loss of neuron structure or functions, including Parkinson's disease (PD), Alzheimer's disease (AD), Huntington's disease (HD), etc.^[^ ^214^ ^]^ These diseases are increasingly being realized to have similar molecular and cellular mechanisms, mainly related to protein misfolding.^[^ ^215^ ^]^ The misfolded proteins aggregate spontaneously and initiate a series of events that ultimately lead to neuronal damage and death.^[^ ^150^ ^]^ Bioactive nanomaterials with unique surface properties can (1) regulate the protein‐protein interaction to inhibit protein aggregation or (2) regulate protein‐cell interactions to remove already formed aggregations.

#### 3.3.1 Inhibit protein aggregation

**FIGURE 9:**
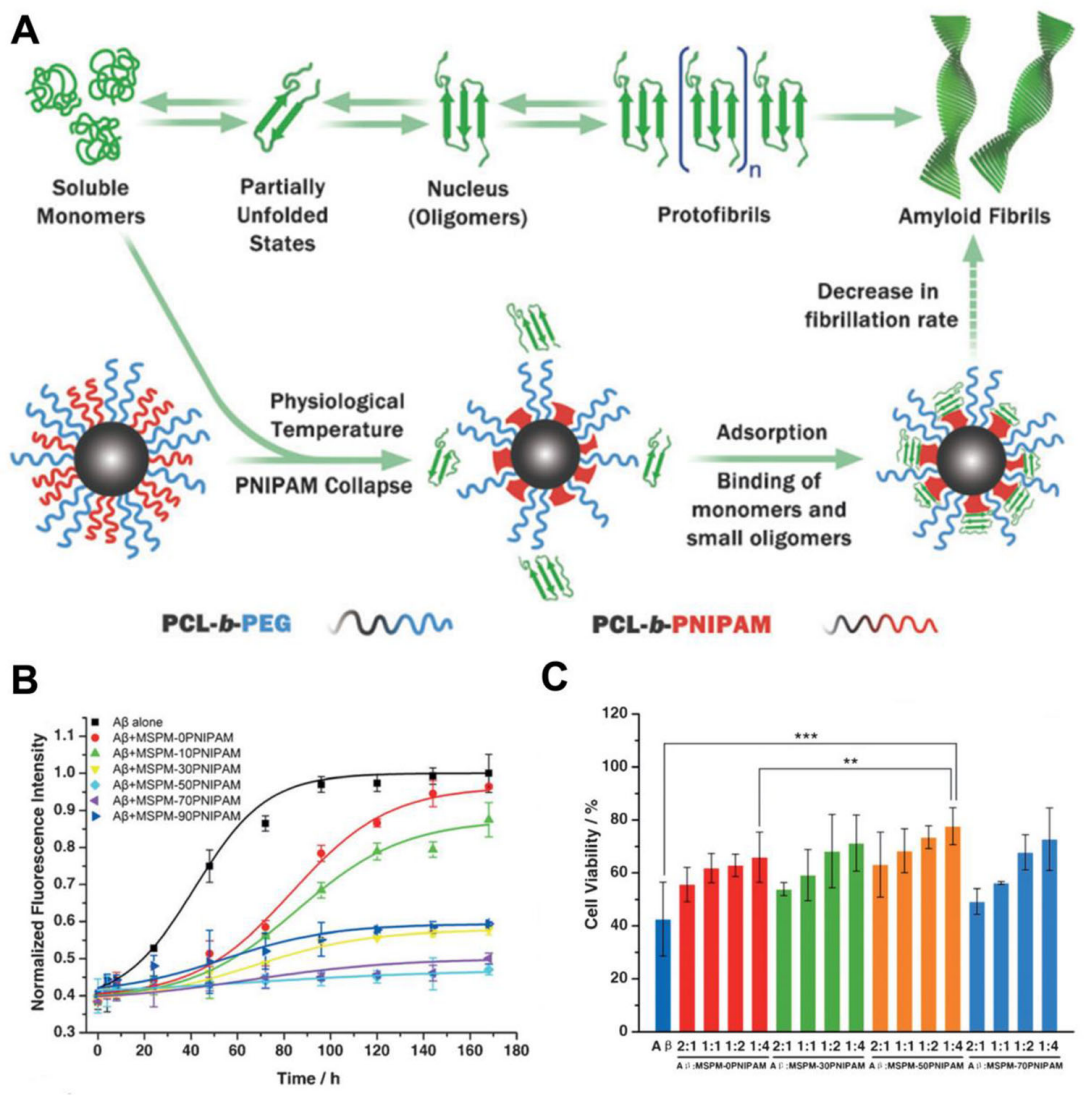
MSPMs for the maintenance of Aβ Homeostasis. (A) Schematic representation of MSPMs to inhibit Aβ aggregation. (B) Fibrillation kinetics of Aβ in the presence of MSPMs with different PCL‐*b*‐PEG/PCL‐*b*‐PNIPAM ratios. (C) Cytotoxicity of four different MSPMs (MSPM‐0, 30, 50, 70 PNIPAM) against PC12 cells. Adapted with permission.^[^ ^150^ ^]^ Copyright 2014, John Wiley & Sons

**FIGURE 10:**
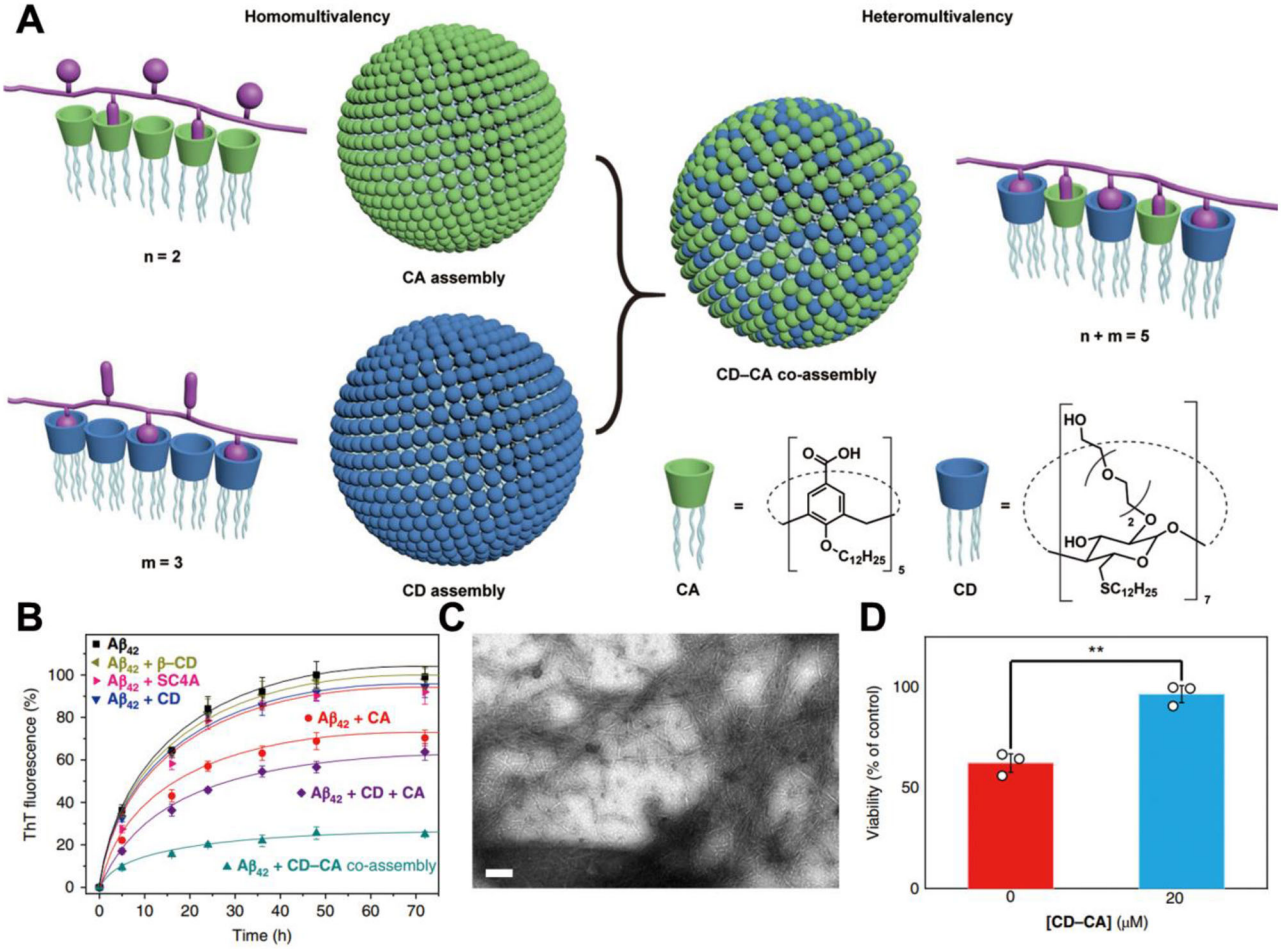
CD‐CA co‐assembly inhibits the formation of Aβ aggregation. (A) Schematic illustration of the heteromultivalent peptide recognition by co‐assembly of CD and CA amphiphiles. (B–D) CD‐CA co‐assembly efficient inhibits the formation of Aβ aggregation and reduces the Aβ‐induced cytotoxicity. Adapted with permission.^[^ ^133^ ^]^ Copyright 2019, Nature Publishing Group

In protein aggregation, the monomeric peptide/proteins (α‐synuclein, β‐amyloid, polyglutamine, Tau) are transformed into partially unfolded intermediates and then aggregate into toxic oligomers.^[^ ^216^ ^]^ This toxic oligomer serves as a template for further monomeric peptide/proteins deposition, which ultimately leads to the formation of insoluble amyloid fibrils. Therefore, by competitively binding with monomer or oligomers, bioactive nanomaterials can effectively inhibit protein aggregation. For example, Shi et al. reported a series of mixed‐shell polymeric micelles (MSPMs) for AD treatment (Figure 9).^[^ ^150^ ^]^ These MSPMs have unique surface phase separation structure formed by hydrophilic chain segments and hydrophobic microdomains. The hydrophobic microdomains act as anchors for hydrophobic Aβ monomers and oligomers, while hydrophilic chain segments create a protective barrier to prevent the further Aβ deposition. As a result, MSPM effectively captured Aβ monomers/oligomers and suppressed the formation of amyloid fibrils. Directly reacting with monomeric peptide/proteins is another feasible strategy to inhibit protein aggregation. For example, Wang et al. presented a reactive conjugated polymer (PPV‐NP) that can covalently react with lysine (K) in Aβ_42_ and provide steric hindrance to inhibit protein aggregation.^[^ ^217^ ^]^ However, these two types of bioactive nanomaterials have a relative weak selectivity to Aβ monomers/oligomers, which limits their in vivo applications. In one recent study, Guo et al. reported a heteromultivalent peptide recognition strategy by co‐assembling calixarene (CA) and cyclodextrin (CD) amphiphiles (CA‐CD) (Figure 10).^[^ ^133^ ^]^ The CA‐CD can simultaneously bind with the tyrosine (Y) and K in β‐amyloid (Aβ_42_), thus improving their specificity to Aβ_42_. Integrating Aβ, Tau, or α‐synuclein‐binding peptides onto bioactive nanoparticles can also be used to improve their specificity to monomers or oligomers. Recently, our group reported a KLVFF (Aβ‐binding peptide)‐integrated bioactive nanocomposite that can specifically bind with Aβ_42_ to inhibit the formation of amyloid fibrils.^[^ ^110^ ^]^ Shi et al. reported a TLK ((D)‐TLKIVW)‐integrated polymeric micelles that can specifically bind with tau aggregation motif (VQIVYK) to inhibit tau protein aggregation.^[^ ^151^ ^]^

#### 3.3.2 Remove already formed aggregations

**FIGURE 11:**
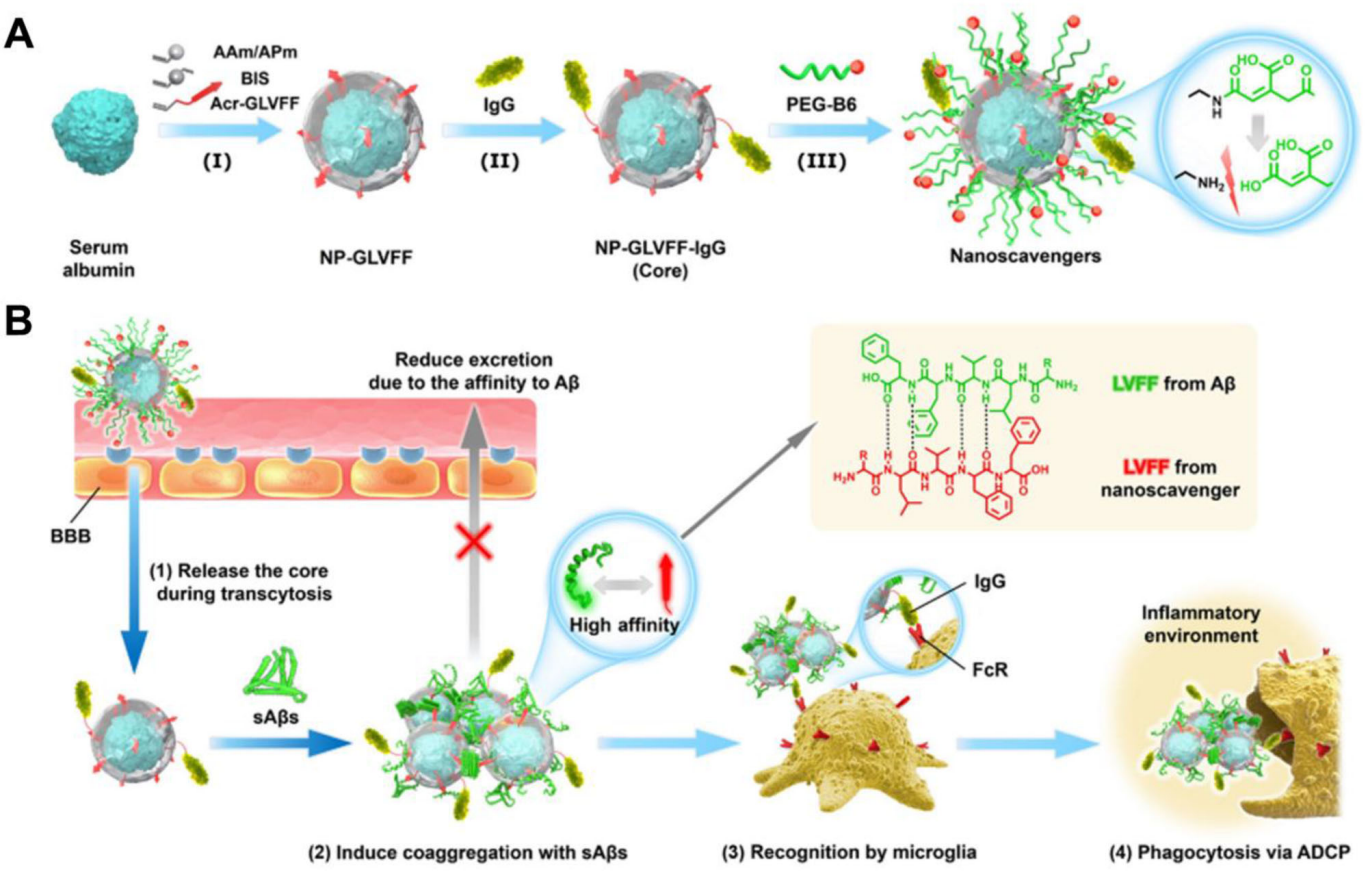
Neuroprotective nanoscavengers facilitate Aβ clearance in AD brain. (A) Schematic for the preparation of nanoscavengers. (B) Schematic diagram of the coaggregation between nanoscavengers and sAβs, and the mechanism of microglia‐mediated clearance of Aβ. Adapted with permission.^[^ ^149^ ^]^ Copyright 2020, Chinese Chemical Society

In pathophysiological brain, the phagocytosis effect of microglia was inhibited by peptide/proteins oligomers and fibrils, resulting in ineffective removal of protein aggregates. Bioactive nanomaterials can effectively regulate the interactions between microglia cells and proteins to promote microglia‐mediated removal of protein aggregations.^[^ ^218^, ^219^, ^220^ ^]^ Recently, Guo et al. constructed a novel Aβ inhibitor by co‐assembling CD with GUA‐modified calixarene (GCA) (denoted as GCA‐CD).^[^ ^156^ ^]^ The GCA‐CD can effectively bind to Aβ42 and form positive‐charged GCA‐CD/Aβ co‐aggregates, thereby promoting the clearance of Aβ aggregates by microglia. Our group reported a neuroprotective nanoscavenger with two functional groups GLVFF (Aβ‐binding peptide) and immunoglobulin G (IgG) on its surface (Figure 11).^[^ ^149^ ^]^ The GLVFF can effectively bind with monomeric Aβ or aggregations and forms Aβ/nanoscavenger co‐aggregates, whereas IgG can facilitate microglia‐mediated clearance of co‐aggregates via activating antibody‐dependent cell‐mediated phagocytosis (ADCP). With the precisely controlled surface property, this neuroprotective nanoscavenger successfully cleared the β‐amyloid aggregations within the brain and improved the cognitive behavior of AD mice.

**FIGURE 12:**
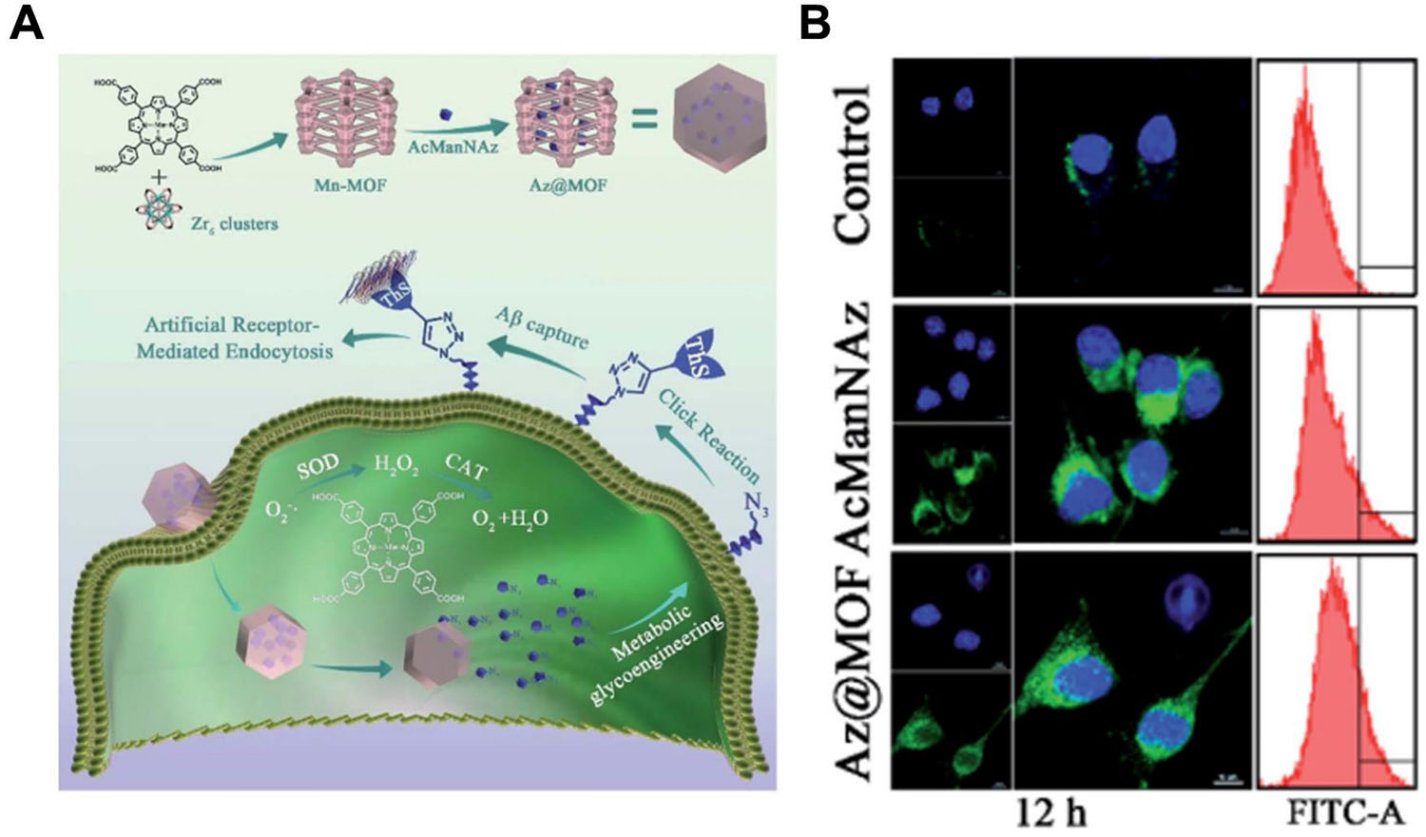
Az@MOF facilitates microglia‐mediated Aβ clearance by constructing artificial receptors. (A) Schematic for the preparation of Az@MOF and its process in promoting microglia‐mediated Aβ clearance. (B) Fluorescence images of intracellular Aβ after pretreatment with PBS, AzManNAz, and Az@MOF (scale bar: 10 mm). Adapted with permission.^[^ ^147^ ^]^ Copyright 2021, The Royal Society of Chemistry

In addition to the bridging effect, bioactive nanomaterials can also form artificial receptors on the microglia surface to regulate their interactions. For example, Qu et al. constructed an artificial receptor (ThS, a thioflavin dye that can selectively capture Aβ aggregates) on microglia (Figure 12).^[^ ^147^ ^]^ The engineered microglia can actively bind to Aβ aggregates and effectively remove Aβ aggregates. Moreover, several carbon‐based nanomaterials, including single‐walled carbon nanotubes, graphene oxide,^[^ ^221^ ^]^ and C60 fullerene derivates,^[^ ^222^ ^]^ can activate the autophagy pathway in microglia, thereby promoting the removal of protein aggregates. However, almost all these bioactive nanomaterials are administrated intracranially due to blood‐brain barrier (BBB), which greatly increases the treatment risk and patient suffering. Therefore, the development of novel strategies for crossing BBB is essential for the safe and efficient treatment of neurodegenerative disease.

### 3.4 Biocatalyst

Biocatalysis refers to the chemical transformation process catalyzed by enzymes or biological organisms (cells, organelles, tissues, etc.), also known as biological transformation.^[^ ^223^, ^224^ ^]^ Over the past few decades, a great number of bioactive materials have been developed to mimic the structure and function of naturally biocatalyst.^[^ ^225^, ^226^, ^227^ ^]^ Among them, nanomaterials with natural enzyme‐like properties have attracted much attention (namely nanozymes). In this section, we will discuss the recent advances in the development of nanozymes in biosensing, imaging, and disease treatment.

#### 3.4.1 Biosensing

Biosensing refers to the conversion of chemical or biological species into measurable signals.^[^ ^228^ ^]^ Colorimetry is the most widely used method in biosensing by employing peroxidase as transducer to catalyze the oxidization of colorless peroxidase substrates into colored products.^[^ ^229^ ^]^ Since Yan group first reported the intrinsic peroxidase‐mimicking capabilities of Fe_3_O_4_ magnetic nanoparticles (MNPs) in 2007,^[^ ^142^ ^]^ dozens of vanadium, noble metal, carbon, and MOF‐based nanozymes have been found to exhibit similar mimicry properties in the following ten years.^[^ ^230^ ^]^ These nanozymes exhibited several advantages such as higher catalytic activities, low‐cost, and physical/chemical stability compared with natural enzymes, thereby offering nanozymes with great potential in biosensing.

**FIGURE 13:**
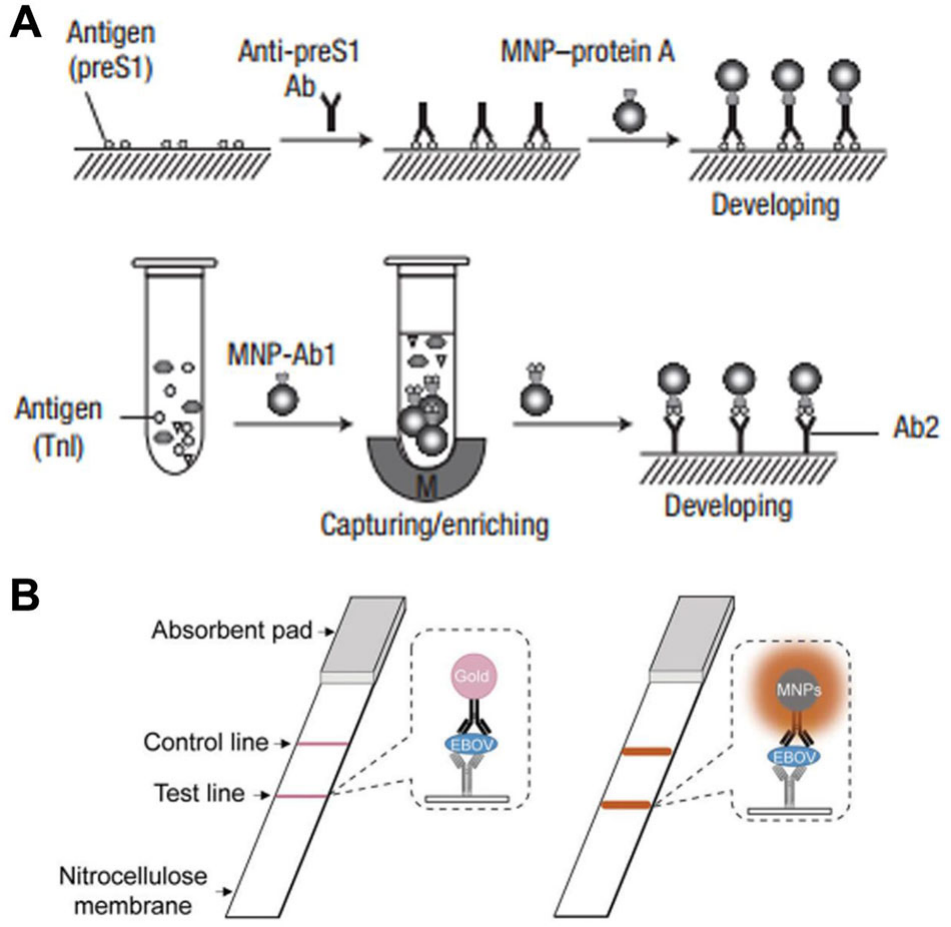
Immunoassays based on the Fe_3_O_4_ MNP. (A) Schematic illustration of Fe_3_O_4_ MNP‐based capture–detection immunoassay. (B) Schematic illustration of Fe_3_O_4_ MNP nanozyme‐strip for the detection of EBOV. (A) Adapted with permission.^[^ ^142^ ^]^ Copyright 2007, Nature Publishing Group. (B) Adapted with permission.^[^ ^143^ ^]^ Copyright 2015, Elsevier

**FIGURE 14:**
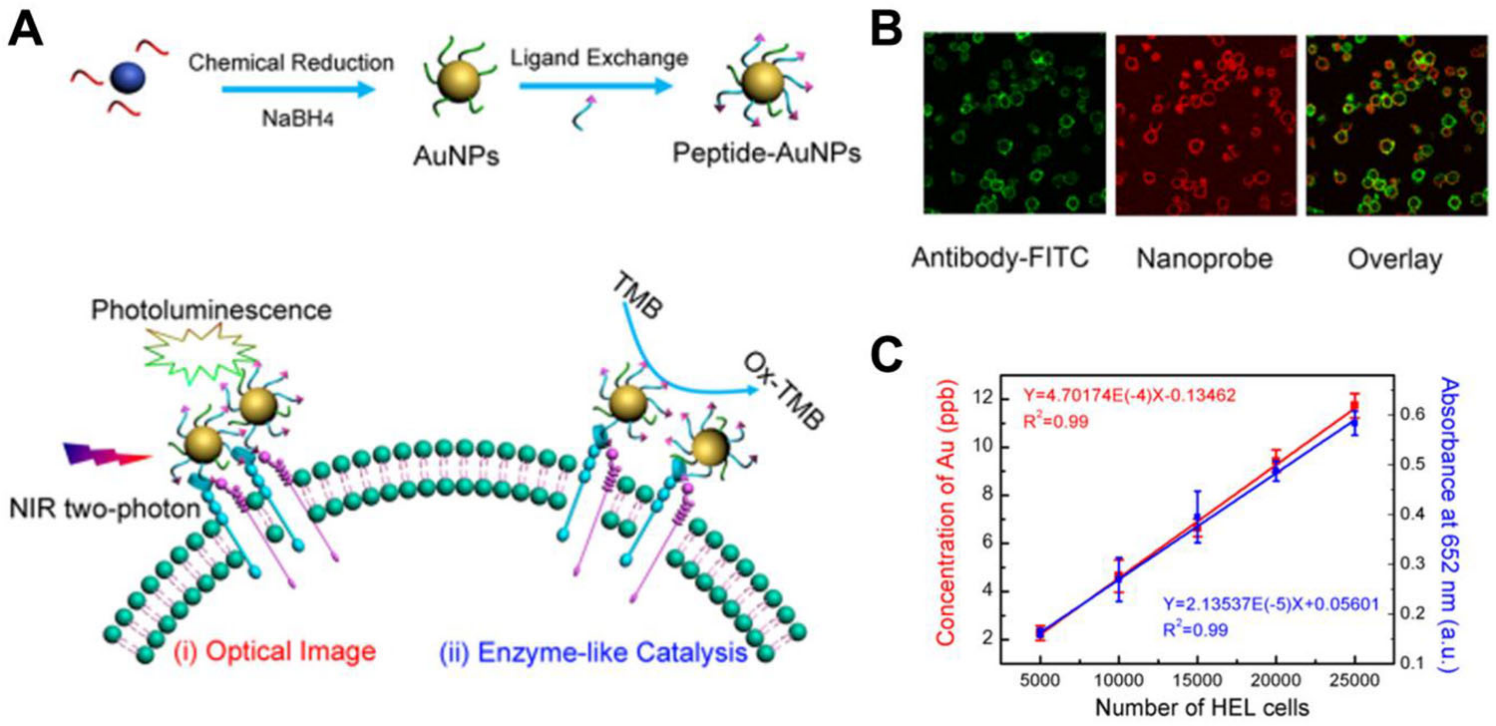
Peptide conjugated AuNP for cancer cell immunoassay. (A) Schematic of peptide‐AuNPs to quantify the expression levels of integrin GPIIb/IIIa on cell surface. (B) peptide‐AuNPs can specifically bind to integrin GPIIb/IIIa. (C) Linear regression associates cell number with catalytic colorimetric variation and Au concentration. Adapted with permission.^[^ ^141^ ^]^ Copyright 2015, American Chemical Society

Nanozymes are capable to detect different chemical and biologic species by combining with different receptors. For example, as combined with oxidases, nanozymes can effectively detect their substrates. By employing glucose oxidase (GOx) as the receptor, Wei et al. reported a ZIF‐based nanozyme GOx/hemin@ZIF‐8 that can effectively detect the glucose in drinks, blood, and urine.^[^ ^146^ ^]^ By employing cholesterol oxidase as receptor, Xu et al. reported a copper sulfide‐based nanozyme BNNS@CuS that can visually detect the total cholesterol in human serum.^[^ ^145^ ^]^ In addition, nanozymes are capable of detecting targeted antigens. In a seminal work, Yan groups integrated the two characteristics of Fe_3_O_4_ nanozyme, peroxidase, and magnetism, and reported a novel capture–detection immunoassay (Figure 13).^[^ ^142^, ^143^ ^]^ This antibody conjugated Fe_3_O_4_ nanozyme can specifically bind to antigen in mixtures, then separates the antigen from the sample under magnet field and generate a colorimetric signal. By employing anti‐cardiac troponin I (TnI) antibody and anti‐EBOV antibody as receptors, this strategy has achieved to capture, separation, and detection of TnI and EBOV. Recently, they developed a novel Co–Fe@hemin nanozyme, and realized the chemiluminescence immunoassay of SARS‐CoV‐2 antigen in serum by loading anti‐SARS‐CoV‐2 antibody.^[^ ^231^ ^]^ Furthermore, nanozymes can detect certain cell phenotype by employing specific cell markers as the targeted antigens. For example, Gao et al. constructed a nanozyme‐based probe to quantify the expression levels of integrin GPIIb/IIIa on cell surface (Figure 14).^[^ ^141^ ^]^ This peptide (H_2_N‐CCYKKKKQAGDV‐COOH) conjugated AuNPs can specially bind to integrin and generate a colorimetric signal. As a result, the expression level of integrin on human erythroleukemia cells can be quantitatively measured in a colorimetric method without cell lysis and protein extraction. Chen et al. reported a platinum NPs/graphene oxide (PtNPs/GO) for cancer cell detection.^[^ ^152^ ^]^ The folic acid (FA) on PtNPs/GO can specifically bind to the FA receptors on cell membrane and in situ generate a colorimetric signal by catalyzing the oxidation of TMB in the presence of H_2_O_2_. In addition to protein receptors, other characteristic markers like epithelial cell adhesion molecule (EpCAM) and glycans can be utilized for cell detection by coupling with their biological recognition ligands such as anti‐EpCAM aptamer (SYL3C) and lectin.^[^ ^232^, ^233^ ^]^

#### 3.4.2 Imaging

**FIGURE 15:**
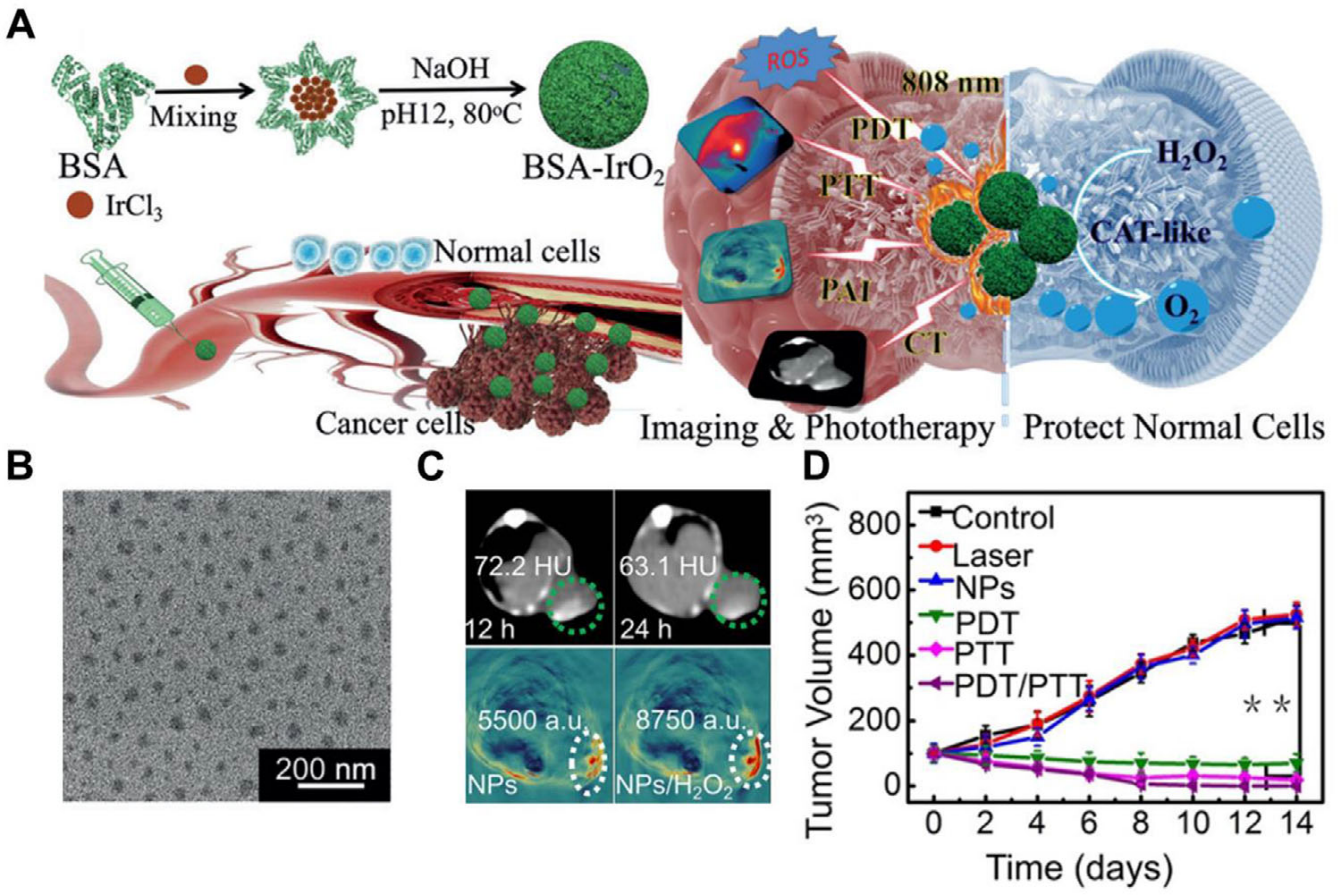
BSA‐IrO_2_ NP for tumor theranostics. (A) Schematic illustration of BSA‐IrO_2_ NPs for efficient tumor theranostics. (B) TEM images of BSA‐IrO_2_. (C) CT and PA images of tumor tissues. (D), BSA‐IrO_2_ can effectively inhibit tumor growth through PDT/PTT. Adapted with permission.^[^ ^144^ ^]^ Copyright 2018, John Wiley & Sons

**FIGURE 16:**
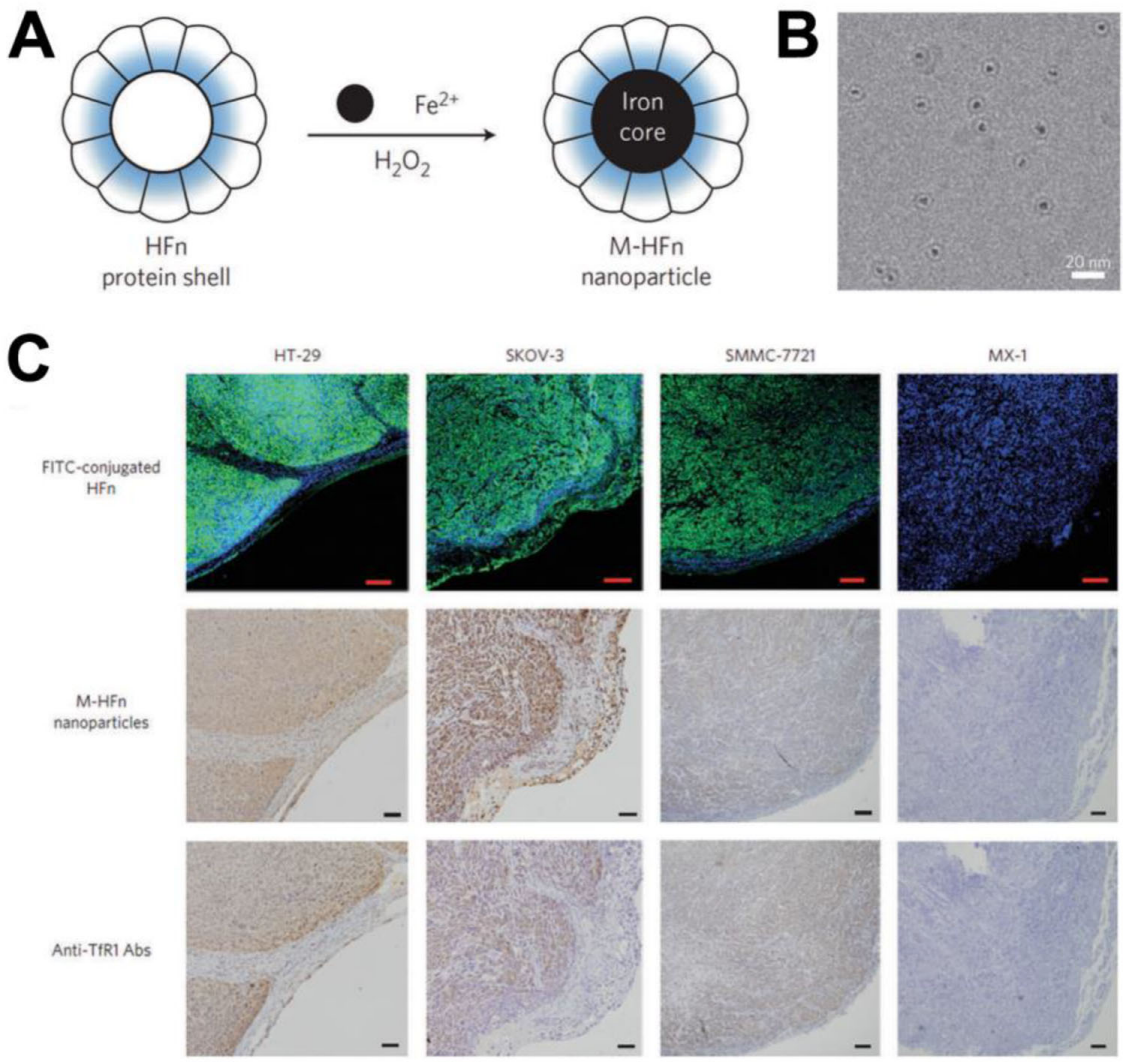
M‐HFn for tumor visualizing. (A) Schematic for the preparation of M‐HFn. (B) TEM images of M‐HFn. (C) M‐HFn as a peroxidase mimic for targeting and visualizing tumor tissues. Adapted with permission.^[^ ^234^ ^]^ Copyright 2012, Nature Publishing Group

Several metal elements have intrinsic properties, for example, iridium (Ir) has X‐ray absorption ability, Fe has magnetism and Au has optics properties. Benefiting from these unique properties, Ir, Fe, and Au‐based nanozymes have been extensively utilized in computed tomography imaging (CT), magnetic resonance imaging (MR), and optical imaging.^[^ ^230^ ^]^ Jiang et al. reported a bovine serum albumin‐iridium oxide nanoparticle (BSA‐IrO_2_ NP) for tumor theranostics (Figure 15).^[^ ^144^ ^]^ BSA‐IrO_2_ NP was prepared via one‐step biomineralization with high X‐ray absorption coefficient, catalysis‐mimicking activity, and extraordinary photothermal conversion efficiency. These characteristics allow BSA‐IrO_2_ NP to achieve efficient CT imaging and treatment of tumors. Yan et al. reported a magnetoferritin NPs (M‐HFn) for tumor targeting and visualization (Figure 16).^[^ ^234^ ^]^ The ferritin (HFn) protein can specifically bind to the transferrin receptor 1 (TfR1) on tumor cells, and loaded iron oxide can catalyze the oxidation of di‐azo‐aminobenzene (DAB) to generate a colorimetric signal and make tumor tissues visualization. Recently, some dual‐modality and multi‐modality imaging strategies have been developed. For example, Cai et al. described a FA‐modified Au nanocluster (FA‐AuNC) for the fluorescence and visualization imaging of tumor tissues.^[^ ^235^ ^]^ The FA‐AuNC can specifically bind to the FA receptors on cell membrane and in situ generate a colorimetric signal for visual imaging, and AuNC can generate an intensive fluorescence signal for fluorescence imaging. As a result, FA‐AuNC provides a molecular colocalization diagnosis for tumor tissues. Similarly, Yang et al. developed a keratin‐templated gold nanocluster (AuNCs@Keratin) for near‐infrared (NIR) fluorescence imaging and MR imaging of tumors.^[^ ^236^ ^]^ These multi‐modality imaging strategies greatly improved the specificity and accuracy of cancer imaging by avoiding false‐negative and false‐positive results.

#### 3.4.3 Disease treatment

**FIGURE 17:**
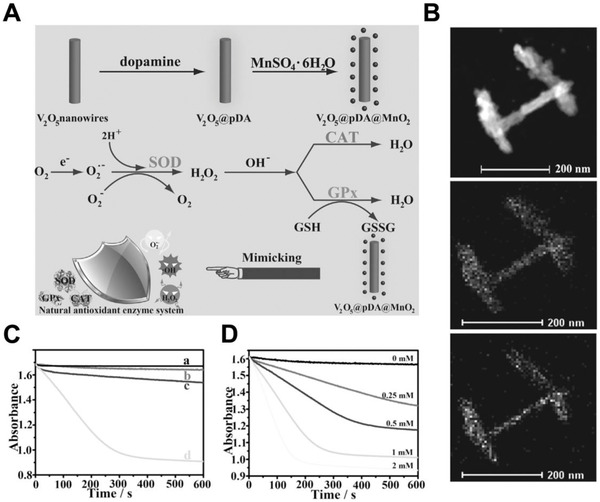
Multi‐nanozyme that mimicking intracellular antioxidant defense system for cytoprotection. (A) Schematic for the synthesis of V_2_O_5_@pDA@MnO_2_ and its process in mimicking intracellular antioxidant‐based defense system to remove ROS. (B) Element compositions analysis with TEM confirmed the formation of V_2_O_5_@pDA@MnO_2_. (C) V_2_O_5_@pDA@MnO_2_ exhibits a GPx‐like activity. (D) V_2_O_5_@pDA@MnO_2_ exhibits a concentration‐dependent oxidize activity to NADPH. Adapted with permission.^[^ ^237^ ^]^ Copyright 2016, John Wiley & Sons

**FIGURE 18:**
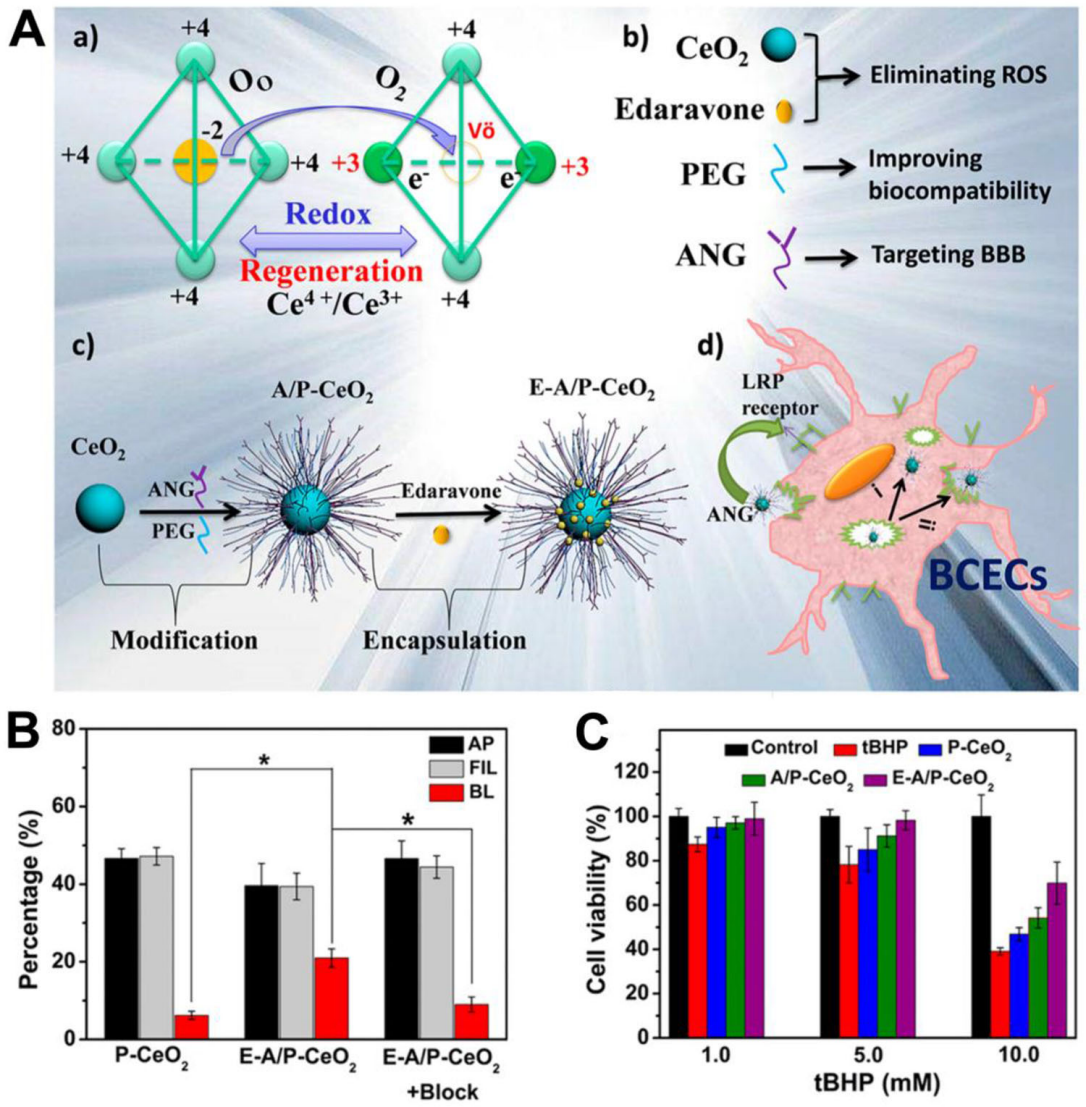
SeO_2_‐based nanozymes (E‐A/P‐CeO_2_) for stroke treatment. (A) Schematic for the synthesis of E‐A/P‐CeO_2_ and its mechanism in crossing BBB and removing ROS. (B) E‐A/P‐CeO_2_ can efficiently cross the BBB. (C) E‐A/P‐CeO_2_ can efficiently remove the ROS. Adapted with permission.^[^ ^238^ ^]^ Copyright 2018, American Chemical Society

Benefiting from their intrinsic catalytic activities, nanozymes also exhibit high performance in disease treatment. Nanozymes with CAT or SOD mimicking activities can scavenge ROS for neuroprotection, cytoprotection, anti‐inflammatory, etc. For example, Qu et al. reported a multi‐nanozyme to mimic intracellular antioxidant defense system for cytoprotection (Figure 17).^[^ ^237^ ^]^ This multi‐nanozyme was prepared by self‐assembling of V_2_O_5_ nanowire with MnO_2_. V_2_O_5_ nanowire exhibit glutathione peroxidase (GPx)‐like activity and MnO_2_ nanoparticle have a SOD and CAT‐like activity. With such unique characteristics, the MnO_2_ nanoparticle effectively eliminated the as‐generated ROS, thereby achieving the cytoprotection. Shi et al. constructed a SeO_2_‐based nanozymes (E‐A/P‐CeO_2_) for stroke treatment (Figure 18).^[^ ^238^ ^]^ The angiopep‐2 (TFFYGGSRGKRNNFKTEEY) on E‐A/P‐CeO_2_ can effectively bind to low‐density lipoprotein receptor (LRP) on endothelial cells and assist E‐A/P‐CeO_2_ to cross BBB. After entering brain tissues, CeO_2_ and edaravone work synergistically to eliminate ROS for effective neuroprotection. Unlike the ROS elimination, nanozymes with oxidase or peroxidase‐mimicking activities can generate ROS in the catalytic process for efficient antitumor and antibacterial applications. Zhao et al. developed a PEG functionalized molybdenum disulfide nanoflowers (PEG‐MoS_2_ NFs) for wound antibacterial applications.^[^ ^239^ ^]^ PEG‐MoS_2_ NFs exhibit both peroxidase‐mimicking activity and high NIR absorption that can effectively convert H_2_O_2_ into ·OH and generate a photothermal effect upon irradiation for synergetic antibacterial effect. Qu et al. presented a Pt nanozyme decorated Zr‐MOF (PCN‐224‐Pt) to relieve tumor hypoxia for enhanced photodynamic therapy (PDT).^[^ ^240^ ^]^ The Pt nanozyme have a CAT‐like activity, which can effectively oxidize H_2_O_2_ into O_2_, thus enhancing the antitumor effect of PDT. In addition, nanozymes can also use for attenuating the drug abuse and addiction by oxidizing neurochemical transmitter, especially dopamine. For example, Xue et al. reported a single‐walled carbon nanotube (SWNTs)‐based nanozyme for the treatment of methamphetamine (METH) addiction.^[^ ^153^ ^]^ Through the adsorption and oxidation of dopamine, SWNTs dramatically attenuated METH‐induced increasing in synaptic protein and tyrosine hydroxylase, ultimately counteracting the goal‐directed behaviors associated with drug addiction.

## 4 SUMMARY AND PERSPECTIVES

Bioactive nanomaterials are a class of biomaterials with nanoscale size that can induce the biological response upon interacting with proteins, cells, or tissues. The bioactivities of bioactive nanomaterials are influenced by numerous factors, including the physical structure of materials, surface property, and nanotopography. These factors have important effects on the interactions between nanomaterials and biological systems, thereby inducing various biological responses. Over the past decade, numerous bioactive nanomaterials have been developed for the treatment of different diseases and tissue regeneration due to their unique bioactivities. For example, inorganic nanomaterials are promising candidates as the bone graft substitutes due to their excellent mechanical strength and antibacterial activity (Section 2.2.1). Polymeric nanomaterials can be easily designed to achieve the tailor‐made structures or customized surfaces for molecular recognition, thereby showing the potentials of modulating the protein‐protein, protein‐cell, and cell‐cell interactions, as well as drug delivery (Section 2.2.2). Moreover, bioactive natural polymers can be directly used to construct drug delivery systems due to their inherent targeting properties. Carbon‐based nanomaterials (e.g., carbon nanotubes, graphene, and graphene oxide) have been widely investigated for bone regeneration and regulation of cellular behaviors such as autophagy and inflammation (Section 2.2.3). The dynamic property and adaptive behavior of noncovalent bonding allow for the convenient dissociation and reconstruction of supramolecular‐based nanomaterials, which open up a wide range of possibilities in the development of deformable nanomedicines/nanocarriers and specific molecular recognition technology (Section 2.2.4). Additionally, carbon nanomaterials and metal‐based nanomaterials have been found to exhibit excellent catalytic activities to mimic natural enzymes. Such abundant bioactivities and biomedical applications benefit from the unique physicochemical properties of bioactive nanomaterials and their tunable nanostructures.

To date, bioactive nanomaterials have been widely investigated for a board range of biomedical applications. However, several challenges still restrict the development and wide application of bioactive nanomaterials, and more efforts should be made in the following aspects.

1. Further studies on chemical mechanisms. Current efforts mainly focus on the development of attractive bioactive nanomaterials and exploring their potential applications. However, the chemical mechanisms in material design have seldom been investigated, especially for inorganic nanomaterials and carbon‐based nanomaterials. Further studies on chemical mechanisms can help researchers to better understand the structure‐activity relationship, thereby providing a guidance for the rational design and development of ideal bioactive nanomaterials.
2. Expanding the scope of bioactive nanomaterials. Researchers usually focus on exploring the bioactivities of traditional nanomaterials, which have been summarized in this review. In recent years, more and more biomaterials with precise nanostructure have been developed, especially DNA‐based materials.^[^ ^241^, ^242^ ^]^ Future perspectives should focus on studying the physicochemical properties and bioactivities of these novel biomaterials. The nanomaterials prepared by 3D printing technology may be an important bioactive nanomaterial, and their potential bioactivities should be evaluated. In addition, the bioactivities of natural nanomaterials also should be explored, due to their excellent biocompatibility and easily accessible sources.
3. Improving the therapeutic efficacy in vivo. Various types of bioactive nanomaterials have been demonstrated by chemists and material specialists in the past few decades. However, their therapeutic efficacy is usually not satisfactory due to the complexity of biological systems. Moreover, A series of issues that must be addressed include the selectivity and efficiency in targeting, biodistribution, biodegradation, and immune response at the organ and system levels.
4. Clinical translational research. Clinical applications, which require collaborative efforts of multidisciplinary experts including those working in material sciences, life sciences, medical sciences, and pharmacy, are seldom studied. For example, the acute and chronic toxicity of bioactive nanomaterials should be further evaluated. In addition, their scale‐up preparation, sterilization, and storage, which are essential for clinical practice, should be focused on.

## CONFLICT OF INTEREST

The authors declare no conflict of interest.
